# Structure‐Guided Engineering of a Cas12i Nuclease Unlocks Near‐PAMless Genome Editing

**DOI:** 10.1002/advs.202516670

**Published:** 2026-01-14

**Authors:** Qitong Chen, Hanlin Gou, Chao Xu, Sihan Wang, Huitao Zhang, Minglei Song, Mengge Wang, Xingkun Ji, Xiaofei Wei, Yuanyan Tan, Hehua Quan, Pengyu Luo, Hanyu Shou, Pengpeng Liu, Yafeng Liang, Jian‐Kang Zhu

**Affiliations:** ^1^ Institute of Advanced Biotechnology Institute of Homeostatic Medicine and School of Medicine Southern University of Science and Technology Shenzhen China; ^2^ Zhejiang University–University of Edinburgh Institute Zhejiang University Hangzhou China

**Keywords:** Cas12i, genome editing, high specificity, PAMless

## Abstract

The therapeutic and research applications of CRISPR‐Cas nucleases are constrained by their reliance on specific Protospacer Adjacent Motifs (PAMs), which limit the accessible sites in the genome. To overcome this critical barrier, we performed structure‐guided engineering of SF01, a compact Cas12i nuclease. Using AlphaFold‐predicted structural models, we identified and systematically mutagenized 38 residues at the PAM‐interacting interface. This iterative engineering process yielded three superior variants—KR, IKRR, and STKRR—that exhibit dramatically relaxed PAM specificity, enabling efficient editing at a broad spectrum of 5'‐NNTN‐3' sites. Importantly, while the most broad‐spectrum variant (STKRR) shows a trade‐off at canonical sites, the IKRR variant retains high activity at canonical 5'‐NTTN‐3' PAMs while simultaneously enabling efficient editing at 5'‐NNTN‐3' sites. This near‐PAMless activity expands the targetable portion of the genome to over 25%, a four‐fold increase over the parental nuclease. Furthermore, adenine base editors (ABEs) constructed with these variants achieve high‐efficiency editing (∼80%) at endogenous loci with expanded targeting scope. Comprehensive off‐target analysis using GUIDE‐tag and Digenome‐seq revealed that the enhanced on‐target activity of the SF01 variants is not accompanied by a loss of specificity. These engineered nucleases represent a powerful and versatile expansion of the genome editing toolkit, enabling applications previously inaccessible due to PAM constraints.

## Graphical abstract

CRISPR‐Cas nucleases are limited by PAM requirements, restricting genome accessibility. Structure‐guided engineering of the compact Cas12i nuclease SF01 produced three variants with near‐PAMless, enabling efficient editing at diverse 5'‐NNTN‐3' sites. These nucleases expand the editable portion of the human genome more than fourfold, enabling efficient base editing with high specificity across diverse mammalian cell types.

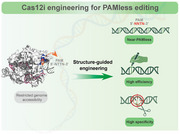

## 1 Introduction

The advent of CRISPR‐Cas technologies has revolutionized the life sciences, providing unprecedented tools for programmable genome editing with profound implications for basic research, biotechnology, and medicine [1, 2]. The most widely adopted systems, derived from Type II Cas9 and Type V Cas12 nucleases, function by using a guide RNA to direct the nuclease to a specific DNA sequence, where it induces a double‐strand break (DSB). However, a fundamental constraint of virtually all natural Cas nucleases is the requirement for a specific DNA sequence immediately adjacent to the target site, known as the Protospacer Adjacent Motif (PAM). This PAM sequence acts as a docking site for the nuclease and is essential for target recognition and cleavage, but its stringent nature severely restricts the genomic loci accessible to editing [3].

Consequently, a major focus of protein engineering has been to broaden the targeting range of Cas nucleases by altering their PAM specificity [4]. While directed evolution and structure‐guided mutagenesis have produced Cas9 [5, 6] and Cas12a [7, 8, 9, 10] variants with relaxed or altered PAM requirements, these expansions often necessitate a trade‐off between targeting range and specificity. For example, SpG and SpRY allow for broad targeting but exhibit lower activity or specificity at certain loci [5, 6]. Similarly, Kleinstiver and colleagues developed enAsCas12a, a variant of *Acidaminococcus sp*. Cas12a with a significantly expanded PAM recognition profile (including TTYN, VTTV, and TRTV) by introducing three key mutations (E174R, S542R, and K548R), which will increase the non‐specific binding affinity of the Cas protein to the DNA backbone. However, this relaxation of PAM stringency in enAsCas12a was accompanied by substantial genome‐wide off‐target activity, necessitating subsequent rounds of engineering to restore fidelity [9, 10]. Furthermore, the large size of Cas12a and Cas9 effectors poses challenges for delivery via size‐constrained vectors such as adeno‐associated viruses (AAV).

In contrast, the Cas12i subfamily member SF01 is a naturally compact nuclease. Here, we report that our structure‐guided engineering of SF01 yields variants that not only achieve near‐PAMless (5'‐NNTN‐3') recognition [11] but, unlike early enAsCas12a variants, retain robust genome‐wide specificity without improved off‐target activity. This combination of a compact form factor, broad targeting range, and high fidelity distinguishes the engineered SF01 variants from previous PAM‐expanded Cas12a tools.

Intriguingly, we observed that the parental SF01 nuclease possesses latent, albeit inefficient, cleavage activity at several non‐canonical PAMs, including ATG‐containing motifs [11]. This intrinsic flexibility suggested an untapped potential for PAM reprogramming. Here, we report the systematic, structure‐guided engineering of SF01 to overcome its PAM limitations. By leveraging structural modeling to inform iterative rounds of mutagenesis at the PAM‐interacting interface, we have developed a suite of SF01 variants that display near‐PAMless activity. These engineered nucleases efficiently target sites with a minimal 5'‐NNTN‐3' PAM while retaining high fidelity and robust on‐target activity, thereby dramatically expanding the targetable genome for a wide array of editing applications.

## 2 Result

### 2.1 Structure‐Guided Mutagenesis of SF01 Enhances Activity on Non‐Canonical PAMs

**FIGURE 1:**
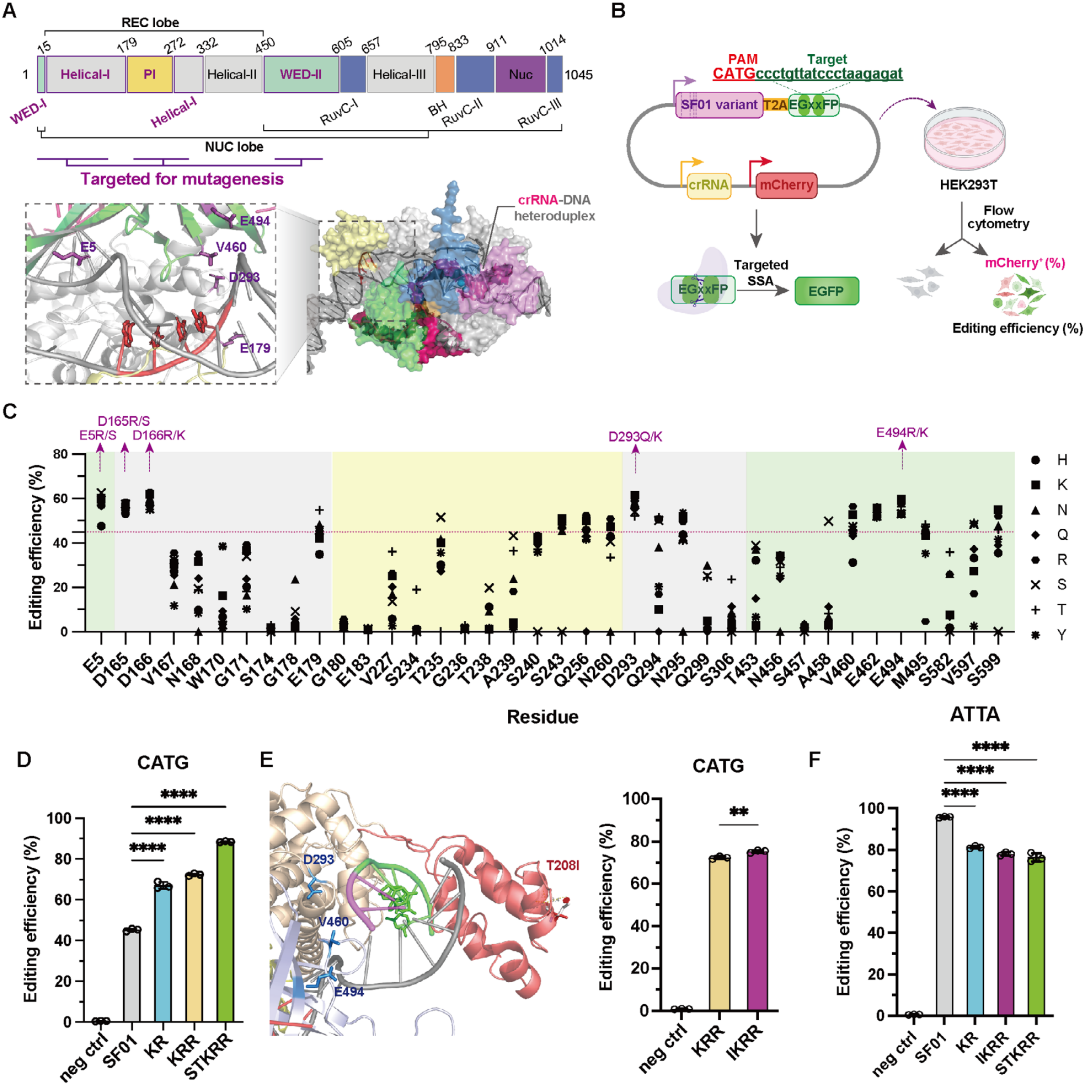
Structure‐guided engineering of SF01 variants with enhanced non‐canonical PAM recognition in HEK293T. (A) Domain architecture of SF01 and AlphaFold‐predicted structure of the SF01‐crRNA‐DNA complex. The inset shows a detailed view of the PAM‐binding interface, highlighting key residues targeted for mutagenesis. (B) Schematic illustration of the EGxxFP reporter system used for activity screening. Nuclease‐induced DSBs at the target site leading to EGFP reconstitution via single‐strand annealing (SSA). (C) Initial activity screening of 38 single‐point mutants at a 5′‐CATG‐3′ PAM site. Ten mutations at five positions were selected for subsequent combinatorial engineering. (D) Editing efficiencies of the top engineered variants (KR, KRR, STKRR) at the CATG PAM. Data are shown as mean ± s.d., n = 3 independent biological replicates. (E) Structural context of residue T208 and the effect of the T208I substitution on the activity of the KRR variant (IKRR). (Left) Structural localization of T208 (red) in the PI domain relative to the KRR mutations (D293K, V460R, E494R). (Right) T208I mildly enhances CATG editing when incorporated into KRR. Data are shown as mean ± s.d., n = 3 independent biological replicates. Statistical significance was evaluated using an unpaired Student's t‐test. (F) Editing efficiency of SF01 and engineered variants at a canonical 5'‐ATTA‐3' PAM site. Data are shown as mean ± s.d., n = 3 independent biological replicates. Statistical significance was evaluated using one‐way ANOVA followed by Dunnett's multiple comparisons test. ^****^ *p* < 0.0001, ^***^ *p* < 0.001, ^**^ *p* < 0.01, ^*^ *p* < 0.05, and ns, not significant.

To expand the targeting range of SF01, we sought to re‐engineer its PAM‐interacting interface. Based on an AlphaFold [12]‐predicted structure, we identified 38 residues across the WED, Helical, and PI domains located in proximity to the PAM duplex (Figure 1A; Table S1). We subjected these sites to semi‐saturation mutagenesis, substituting each with a panel of eight amino acids (H, K, N, Q, R, S, T, Y) [13] selected for their potential to form new interactions with the DNA backbone or bases. Using a split‐EGFP reporter assay dependent on cleavage at a target site with a non‐canonical 5'‐CATG‐3' PAM (Figure 1B), we screened an initial library of 293 single‐mutant variants. This screen identified ten substitutions at five key positions (E5, D165, D166, D293, E494) that consistently enhanced editing activity above the parental SF01 baseline (Figure 1C). This synthetic single‐mutant library enabled systematic exploration of PAM‐interacting residues to expand SF01's targeting range.

We next performed iterative rounds of combinatorial mutagenesis. A screen of 40 double mutants identified D293K_E494R (KR) and D293Q_E494R (QR) as the most active variants (Figure S1A). Further combinations led to the selection of a top‐performing triple mutant, D293K_V460R_E494R (KRR), and a quintuple mutant, E5S_E179T_D293K_V460R_E494R (STKRR) (Figure S1B,C). These three variants—KR, KRR, and STKRR—demonstrated a progressive, up to 2‐fold enhancement in editing efficiency on the CATG PAM compared to the ancestral SF01 (Figure 1D). To synergistically enhance the electrostatic interactions conferred by the KR/R mutations, we introduced a polar‐to‐nonpolar substitution within the PI domain (Figure S2A). We identified an adjuvant mutation, T208I, which structural modeling suggests may reduce PAM stringency by disrupting a local hydrogen‐bonding network (Figure 1E). Incorporation of T208I modestly improved the editing activity of the KRR (hereafter referred to as IKRR) (Figure 1E; Figure S2B). In the reporter assay, compared to the parental SF01, the KR and IKRR variants retained the majority of the parental activity at the canonical PAM (∼95% relative to SF01), whereas STKRR showed a reduction to approximately 75%, consistent with a trade‐off often observed in PAM‐relaxed variants (Figure 1F).

### 2.2 Engineered SF01 Variants Exhibit Robust Editing at Endogenous Loci

**FIGURE 2:**
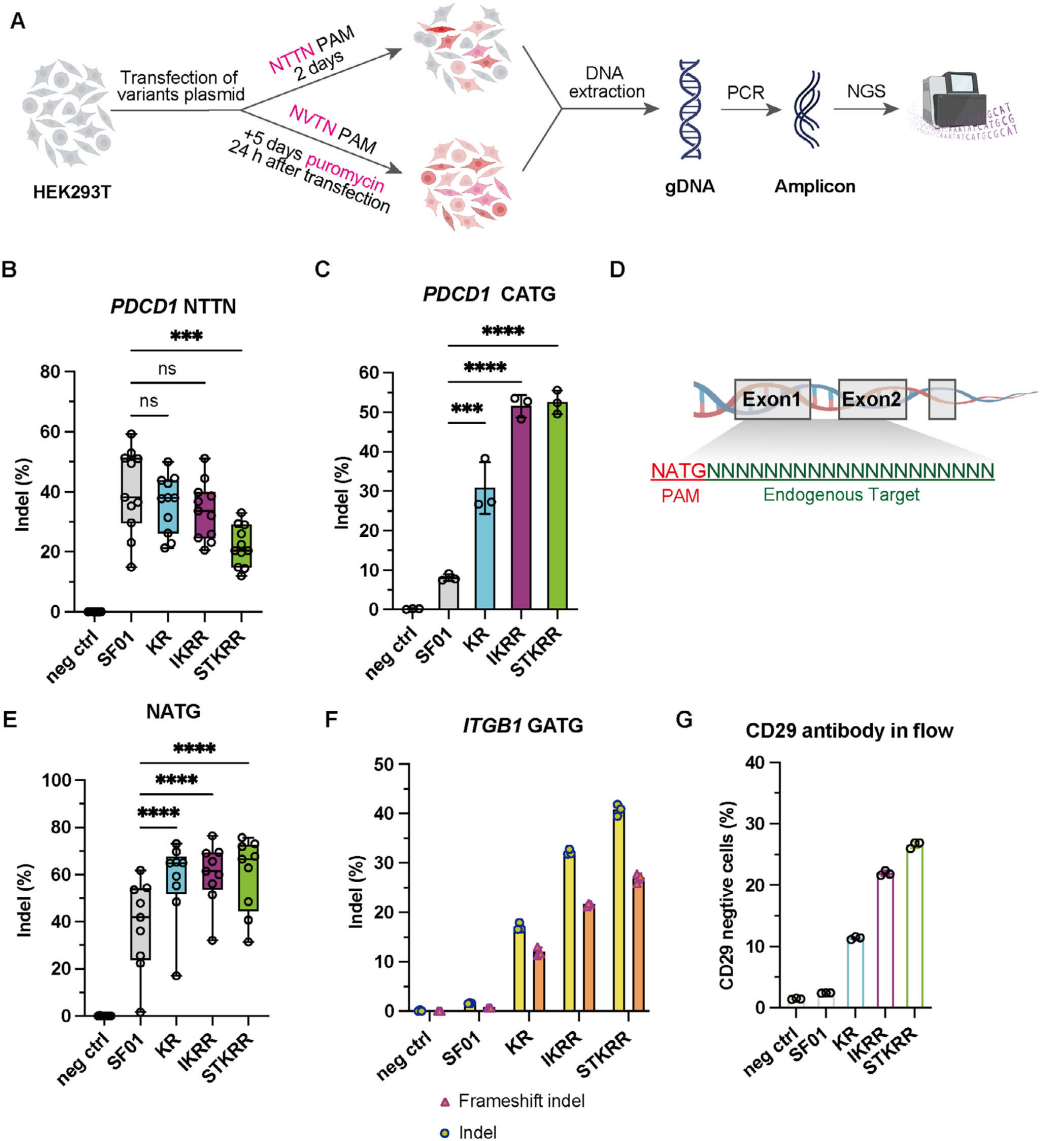
Robust editing by SF01 variants at endogenous genomic loci in HEK293T cells. (A) Experimental workflow for assessing editing at canonical (NTTN) and non‐canonical (NNTN) PAM sites. (B) Box plot summarizing indel efficiencies of SF01 and variants at 11 endogenous sites with canonical NTTN PAMs in the *PDCD1* gene (see Figure S3). Data are shown as mean ± s.d., n = 3 independent biological replicates. Statistical significance was assessed using two‐way ANOVA to compare all group means. (C) Indel efficiencies at an endogenous *PDCD1* locus containing a CATG PAM. Data are shown as mean ± s.d., n = 3 independent biological replicates. Statistical significance was evaluated using one‐way ANOVA followed by Dunnett's multiple comparisons test. (D) Schematic illustration of endogenous target sites with ATG PAMs. (E) Box plot summarizing indel efficiencies at multiple endogenous NATG PAM sites (see Figure S4). Data are shown as mean ± s.d., n = 3 independent biological replicates. Statistical significance was assessed using two‐way ANOVA to compare all group means. (F) Indel and frameshift frequencies at the *ITGB1* (CD29) locus (GATG PAM). Data are shown as mean ± s.d., n = 3 independent biological replicates. (G) Flow cytometry analysis of CD29 expression following genome editing. Data are shown as mean ± s.d., n = 3 independent biological replicates. ^****^ *p* < 0.0001, ^***^ *p* < 0.001, ^**^ *p* < 0.01, ^*^ *p* < 0.05, and ns, not significant.

To validate these findings in a native chromatin context, we assessed the editing efficiencies of SF01 and the top variants (KR, IKRR, STKRR) at endogenous loci in HEK293T cells (Figure 2A). At 11 sites within the *PDCD1* gene bearing canonical 5'‐NTTN‐3' PAMs, including ATTG, CTTC (site1‐7), TTTG, TTTA, and GTTG, the target sequences are listed in Table S2. Overall, at canonical 5'‐NTTN‐3' sites, the KR and IKRR variants displayed activity levels nearly identical to wild‐type SF01. In contrast, STKRR exhibited a notable reduction (approximately 50%), suggesting that IKRR serves as the optimal variant when preserving canonical activity is a priority, while STKRR is best reserved for difficult‐to‐target non‐canonical sites (Figure 2B; Figure S3). In stark contrast, at a *PDCD1* site containing a non‐canonical 5'‐CATG‐3' PAM, all variants showed dramatically improved performance, with STKRR achieving an indel frequency of 52.5%, a 6.5‐fold enhancement over the parental SF01 (Figure 2C).

We next tested a broader range of non‐canonical 5'‐NATG‐3' PAMs at endogenous loci within several therapeutically relevant genes, including *PCSK9*, *TTR*, and *VEGFA* (Figure 2D). The engineered variants consistently and robustly edited these sites, far outperforming the wild‐type enzyme (Figure 2E; Figure S4). To confirm that this high editing activity translates to a functional outcome, we targeted the *ITGB1* gene (encoding the cell surface marker CD29) at a site with a 5'‐GATG‐3' PAM. Targeted deep sequencing confirmed high indel frequencies, with the STKRR variant inducing the most frameshift mutations (Figure 2F). Correspondingly, flow cytometry analysis revealed a marked reduction in CD29 surface expression, with 26.8% of STKRR‐edited cells becoming CD29‐negative, compared to only 2.4% for SF01 (Figure 2G; Figure S5).

### 2.3 Systematic Profiling Reveals a Near‐PAMless 5'‐NNTN‐3' Recognition Landscape In Vitro and In Mammalian Cells

**FIGURE 3:**
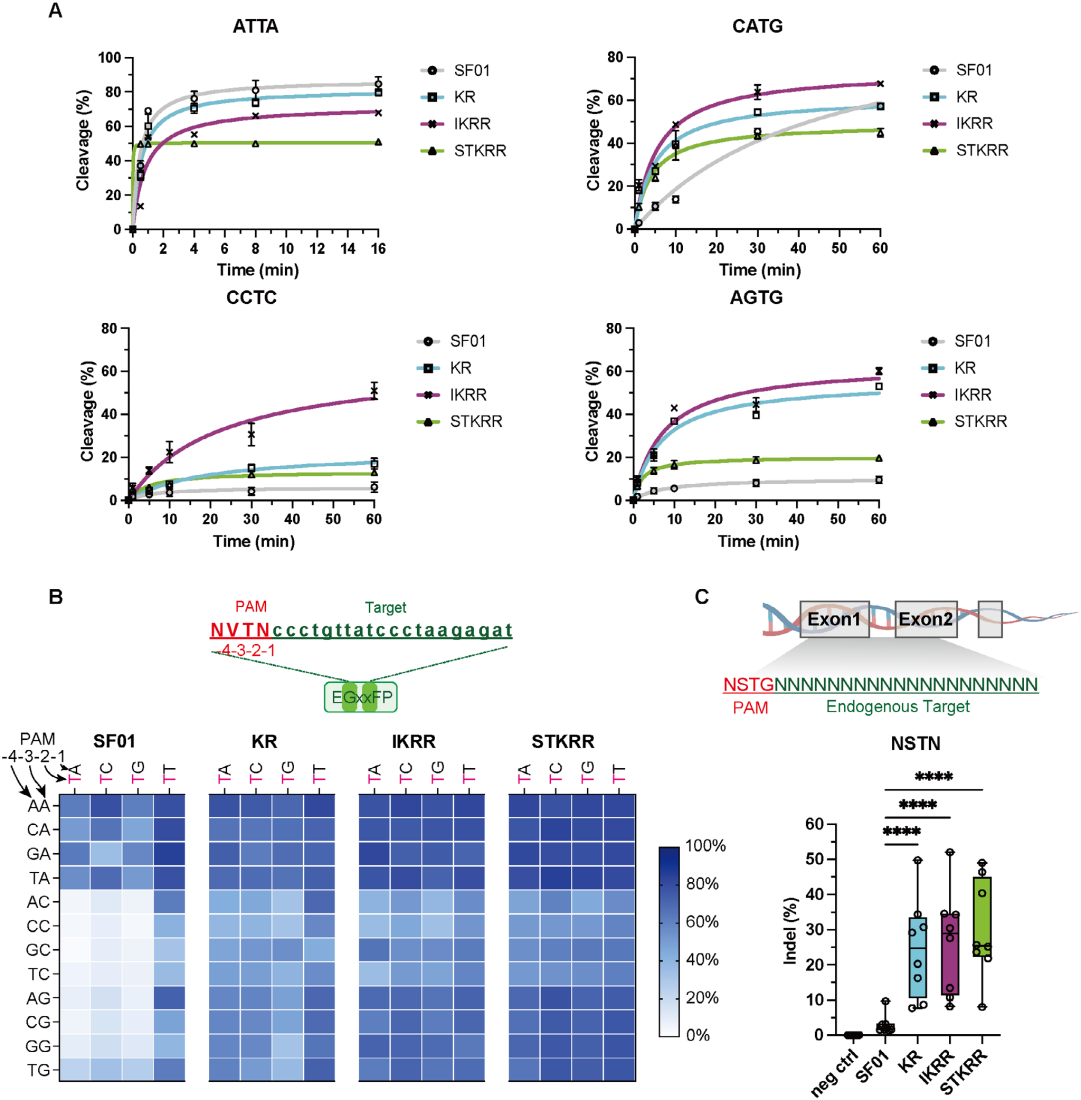
Engineered SF01 variants exhibit an expanded, near‐PAMless recognition profile in vitro and in HEK293T cells. (A) Time‐course DNA cleavage assays were performed for SF01, KR, IKRR, and STKRR using substrates containing different PAM sequences. The kinetic curves were fit to the Michaelis–Menten equation. Data are shown as mean ± s.d., n = 3 independent biological replicates. (B) (Top) Schematic illustration of the EGxxFP reporter system constructed to evaluate activity at 5’‐NVTN‐3’ PAM. (Bottom) Heatmap showing the editing efficiencies of SF01 and variants across all 16 5'‐NVTN‐3' PAMs in the reporter assay. Data are shown as mean ± s.d., n = 3 independent biological replicates. (C) Schematic illustration of endogenous target sites with diverse 5'‐NSTN‐3' PAMs (top) and box plots summarizing indel efficiencies at multiple endogenous NSTN PAM sites (bottom; see Figure S7). Data are shown as mean ± s.d., n = 3 independent biological replicates. Statistical significance was assessed using two‐way ANOVA to compare all group means. ****p < 0.0001, ***p < 0.001, **p < 0.01, *p < 0.05, and ns, not significant.

The SF01 and its variants (KR, IKRR, STKRR) were successfully expressed and purified (Figure S6A) to biochemically validate their PAM recognition preferences. Enzyme activity assays confirmed that all the purified proteins exhibited robust cleavage activity using the *DNMT1‐*containing PCR product (Figure S6B). To further explore the kinetic properties, we performed detailed cleavage assays with synthetic dsDNA substrates (Table S2) bearing various PAM sequences. All three variants showed cleavage rates comparable to the SF01 when tested with a canonical 5′‐ATTA‐3′ PAM‐containing substrate. Interestingly, STKRR displayed strong binding affinity, enabling rapid substrate binding and cleavage; however, its cleavage activity was slightly reduced compared to SF01 when using the 5′‐ATTA‐3′ PAM. For non‐canonical PAMs, such as 5′‐CATG CCTC AGTG‐3′, all three variants significantly outperformed SF01, with IKRR showing the highest cleavage efficiency. These results highlight the enhanced PAM compatibility and specificity of the variants, particularly in the context of non‐canonical PAMs (Figure 3A; Figure S6C).

To systematically map the PAM recognition landscape of our engineered variants, we tested their activity against a library of all 16 possible 5'‐NVTN‐3' (V = A or C or G) PAMs in the EGFP reporter system. This analysis revealed a profound expansion of PAM compatibility for KR, IKRR, and STKRR, with strong activity observed across nearly all tested sequences, in contrast to the highly restricted profile of the parental SF01 (Figure 3B). This suggested that the variants' primary requirement had been relaxed to a single thymine at the ‐2 position (5'‐NNTN‐3').

To confirm this near‐PAMless activity at endogenous loci, we selected eight sites with diverse 5'‐NSTN‐3' PAMs (where S = C or G) (Figure 3C; Figure S7). At all tested sites, including those with CCTC, GCTC, TCTG, AGTC, AGTG, CGTA, GGTC, and TGTG PAMs, the three engineered variants demonstrated consistently high editing efficiencies, whereas the parental SF01 was largely inactive. These results confirm that our engineering efforts successfully relaxed PAM preference to a minimal 5'‐NNTN‐3' requirement, substantially expanding the genomic targeting space.

### 2.4 SF01 Variants are Effective Across Diverse Cell Types and Enable Allele‐Specific Editing

**FIGURE 4:**
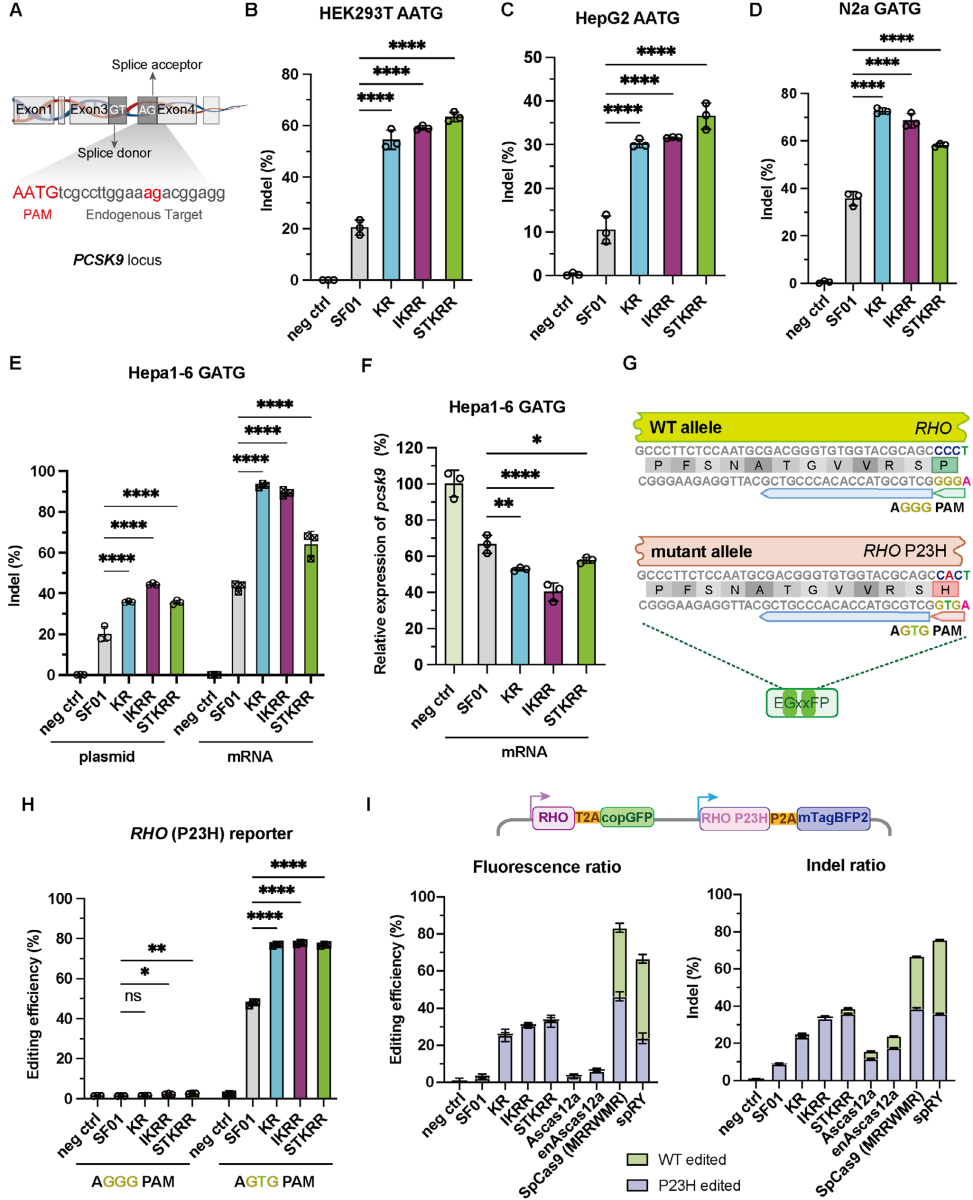
SF01 variants are active across diverse cell types and enable allele‐specific editing. (A) Schematic illustration indicates the selected human *PCSK9* target site within an mRNA splice acceptor region. (B, C) Indel efficiencies at a human *PCSK9* splice acceptor site (AATG PAM) in HEK293T (B) and HepG2 (C) cells. Data are shown as mean ± s.d., n = 3 independent biological replicates. (D) Indel efficiencies at the *PCSK9* start codon (GATG PAM) in mouse N2a cells. Data are shown as mean ± s.d., n = 3 independent biological replicates. (E) Indel efficiencies at the *PCSK9* start codon (GATG PAM) in mouse Hepa1‐6 cells following plasmid or mRNA delivery. Data are shown as mean ± s.d., n = 3 independent biological replicates. (F) Relative *PCSK9* expression level (%) in Hepa1‐6 cells following mRNA‐mediated editing. Data are shown as mean ± s.d., n = 3 independent biological replicates. (G) Schematic illustration of EGxxFP reporter constructs modeling the *RHO* wild‐type and *RHO* P23H alleles. (H) Editing efficiencies of the *RHO* and *RHO* P23H alleles assessed using the EGxxFP reporter assay. Data are shown as mean ± s.d., n = 3 independent biological replicates. (I) Schematic illustration of the generation of a piggyBac‐based stable cell line co‐expressing RHO‐GFP and RHO P23H–BFP (top). Fluorescence‐based editing purity (left) and indel‐based editing purity by NGS (right) of SF01, KR, IKRR, STKRR, AsCas12a, enAsCas12a, SpCas9 (MRRWMR), and spRY (bottom). Data are shown as mean ± s.d., n = 3 independent biological replicates. Statistical significance was evaluated using one‐way ANOVA followed by Dunnett's multiple comparisons test. ^****^ *p* < 0.0001, ^***^ *p* < 0.001, ^**^ *p* < 0.01, ^*^ *p* < 0.05, and ns, not significant.

To assess the generalizability of our variants, we tested their performance in human hepatoma (HepG2) cells, mouse neuroblastoma (N2a), and mouse hepatoma (Hepa1‐6) cells. The variants efficiently edited a splice acceptor site [14] (AATG PAM) in human *PCSK9* in both HEK293T and HepG2 cells (Figure 4A–C), and the *PCSK9* start codon region (GATG PAM) in N2a and Hepa1‐6 cells (Figure 4D,E), demonstrating robust activity across different species, cell types, and genomic contexts. Given the efficient editing of the *PCSK9* start codon region (GATG PAM) in Hepa1‐6 cells, we further investigated the effects on *PCSK9* expression by mRNA electroporation. We observed that both SF01 and its variants significantly reduced *PCSK9* expression (Figure 4F), with the IKRR variant showing the most pronounced effect, achieving a 60% decrease—almost double that of SF01. These results suggest that SF01 and its variants can effectively regulate the expression levels of genes they edit, highlighting their potential for gene expression modulation.

The expanded PAM compatibility of our variants creates new opportunities for therapeutic applications, such as allele‐specific editing. The P23H mutation in the human Rhodopsin (*RHO*) gene [15, 16, 17], a major cause of autosomal dominant retinitis pigmentosa, creates a novel 5'‐AGTG‐3' PAM that is targetable by our variants, while the wild‐type allele contains a non‐targetable 5'‐AGGG‐3' PAM [18] (Figure 4G). Using a reporter assay, we confirmed that KR, IKRR, and STKRR all edited the mutant P23H allele with high efficiency (∼77%), a significant improvement over SF01 (48%). Crucially, none of the nucleases showed any detectable activity on the wild‐type allele, demonstrating near‐perfect allele discrimination (Figure 4H).

Following the demonstration of allele‐specific editing efficiency for the *RHO* allele in the reporter assay, we sought to benchmark the practical performance of our variants against current state‐of‐the‐art PAM‐flexible enzymes in a side‐by‐side study. We targeted the *RHO* P23H locus using our top SF01 variants (KR, IKRR, STKRR) alongside AsCas12a, enAsCas12a [9, 10], the near‐PAMless SpRY Cas9 [19], and a recently published high‐fidelity SpCas9 variant (MRRWMR) optimized for *RHO* targeting [20]. To ensure a rigorous comparison, we generated stable HEK293T cell lines using a piggyBac vector co‐expressing the wild‐type allele (CMV‐RHO‐T2A‐copGFP) and the mutant allele (SFFV‐RHO‐P23H‐P2A‐mTagBFP2). As shown in Figure 4I and Figure S8, the SF01 variants delivered a superior balance of potency and specificity.

Regarding efficiency, the KR, IKRR, and STKRR variants achieved robust editing of the P23H mutant allele (5'‐AGTG‐3' PAM), matching the efficiency of the optimized SpCas9 and significantly outperforming both AsCas12a and enAsCas12a, which showed minimal activity at this locus. Crucially, regarding specificity, the SF01 variants exhibited negligible activity on the wild‐type allele (5'‐AGGG‐3'), demonstrating precise single‐nucleotide discrimination. In sharp contrast, both SpRY and the SpCas9 (MRRWMR) variant displayed high levels of promiscuity, editing the wild‐type allele with frequencies approaching ∼50%. This direct comparison highlights a key advantage of the SF01 5'‐NNTN‐3' recognition mode: unlike the near‐PAMless SpRY, SF01 retains sufficient PAM‐sensing stringency to enable highly specific allele discrimination while still offering a dramatically expanded targeting range compared to Cas12a.

### 2.5 SF01 Variants Serve as a Platform for High‐Efficiency Adenine Base Editing

To broaden the utility of our PAM‐flexible SF01 platform beyond gene disruption, we adapted the variants for precise adenine base editing. First, we generated a catalytically inactive or “dead” SF01 (dSF01) by mutating key residues within the nuclease domain, guided by structural alignment with the homologous Cas12i2 nuclease [21] (Figure S9A). Functional screening confirmed that the D619A and E844A mutations effectively abolished cleavage activity, with the E844A variant selected as the optimal scaffold for its superior performance when fused to a deaminase (Figure S9B,C).

**FIGURE 5:**
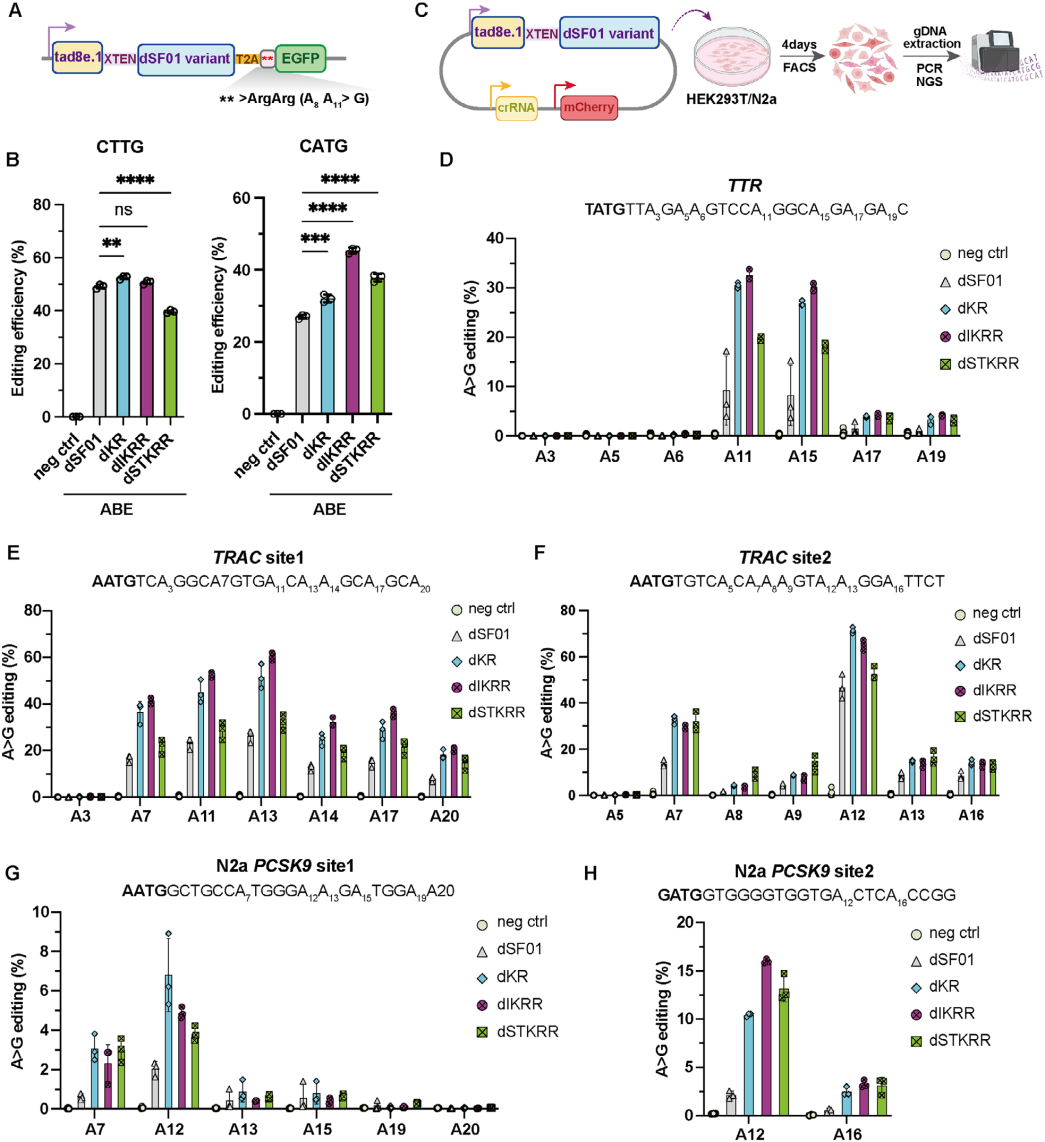
Development of high‐efficiency adenine base editors with expanded targeting scope. (A) Schematic illustration of the fluorescence‐based ABE reporter system. (B) ABE activity of dSF01 and engineered variants at canonical (CTTG) and non‐canonical (CATG) PAM sites in the reporter assay. Data are shown as mean ± s.d., n = 3 independent biological replicates. Statistical significance was evaluated using one‐way ANOVA followed by Dunnett's multiple comparisons test. ^****^ *p* < 0.0001, ^***^ *p* < 0.001, ^**^ *p* < 0.01, ^*^ *p* < 0.05, and ns, not significant. (C) Workflow for assessing ABE activity at endogenous loci. (D–F) A‐to‐G base editing efficiencies by NGS at the *TTR* (D) and *TRAC* (E, F) loci in HEK293T cells. Data are shown as mean ± s.d., n = 3 independent biological replicates. (G, H) A‐to‐G base editing efficiencies by NGS at two *PCSK9* loci in N2a cells. Data are shown as mean ± s.d., n = 3 independent biological replicates.

We then constructed a suite of adenine base editors (ABEs) by fusing the highly active TadA8e.1 [22, 23] deaminase to the dSF01‐E844A scaffold and its engineered derivatives (dKR, dIKRR, dSTKRR). Using a fluorescence‐based reporter that restores EGFP expression upon a targeted A‐to‐G conversion (Figure 5A), we first confirmed that the dKR‐ABE and dIKRR‐ABE variants retained high activity at a canonical 5'‐CTTG‐3' PAM, whereas dSTKRR‐ABE showed reduced efficiency in this context. More importantly, at a non‐canonical 5'‐CATG‐3' PAM site, the engineered variants significantly outperformed the parental dSF01‐ABE, with dIKRR‐ABE showing a 1.6‐fold increase in base editing efficiency (Figure 5B).

To validate these findings at endogenous loci, we deployed the ABEs in HEK293T and N2a cells (Figure 5C). The engineered variants consistently enabled high‐efficiency A‐to‐G editing at non‐canonical PAM sites that were inaccessible to the parental ABE. At the human *TRAC* locus, for example, the dKR‐ABE variant achieved editing efficiencies of up to 72%, while the parental dSF01‐ABE was inactive (Figure 5D–F). Similar enhancements were observed at two *PCSK9* sites in mouse N2a cells (Figure 5G,H). Collectively, these results establish that our engineered SF01 variants serve as a versatile and robust platform for high‐efficiency base editing, capable of precisely targeting previously inaccessible genomic loci.

### 2.6 Comprehensive Profiling Reveals that SF01 Variants Maintain High Genome‐Wide Specificity

A critical consideration when engineering nucleases for broadened PAM compatibility is the potential for increased off‐target activity. To rigorously assess the genome‐wide specificity of our engineered variants, we employed a two‐pronged approach: GUIDE‐tag, a sensitive in‐cellulo method that captures DSB events within the native chromatin context [24, 25], and Digenome‐seq [26, 27], an unbiased in vitro method that digests purified genomic DNA to reveal intrinsic off‐target propensities, to cross‐validate our findings. Both methods were applied to SF01 and its variants (KR, IKRR, STKRR) targeting endogenous sites in both mouse N2a cells (targeting *PCSK9* with a GATG PAM) and human HEK293T cells (targeting *PDCD1* with a GTTG PAM, *PCSK9* with an AATG PAM, and *DNMT1* with a TTTC PAM). As a benchmark for specificity, we also analyzed AsCas12a and its engineered variant, enAsCas12a, targeting *DNMT1*.

**FIGURE 6:**
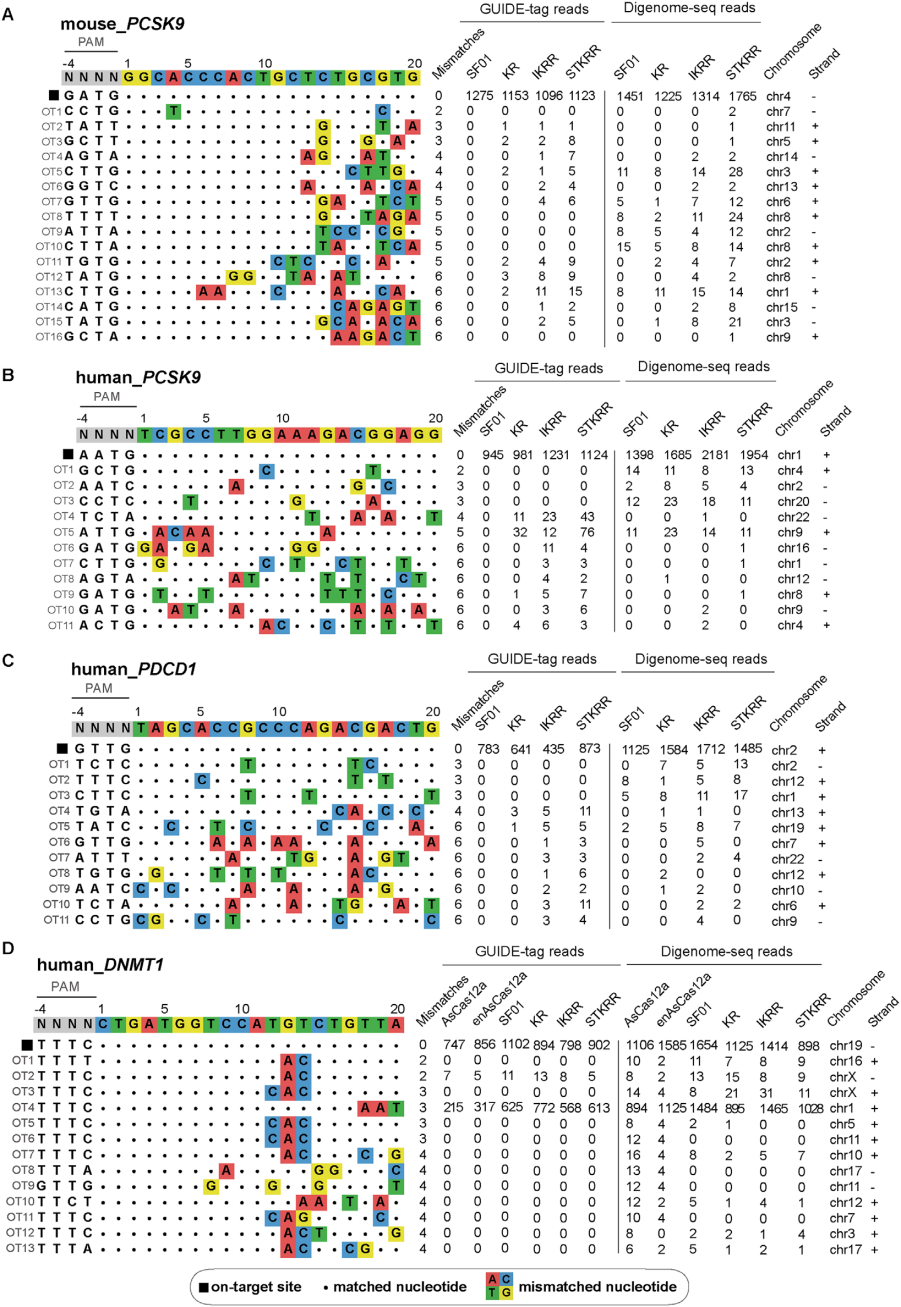
Genome‐wide off‐target profiling of SF01 and its variants using Guide‐tag and Digenome‐seq. (A–C) GUIDE‐tag and Digenome‐seq analysis of SF01 KR, IKRR, and STKRR. (D) GUIDE‐tag and Digenome‐seq analysis of SF01, KR, IKRR, STKRR, AsCas12a, and enAsCas12a. On‐target and identified potential off‐target sequences are shown.

The GUIDE‐tag analysis revealed that while all variants captured a high number of reads at their respective on‐target sites, the engineered variants tended to identify a greater number of potentials off‐target sites in cellulo compared to the parental SF01 (Figure 6A–D). For instance, at the human *PDCD1* locus, the STKRR variant captured more reads at several potential off‐target sites than SF01. These candidate sites typically contained 3 to 5 mismatches relative to the on‐target sequence, suggesting that the introduced mutations may enhance the initial DNA binding affinity of the nuclease even at imperfectly matched sites. However, Digenome‐seq analysis—which strictly measures DNA cleavage—did not show a corresponding increase in off‐target cuts for the variants. Meanwhile, the elevated GUIDE‐tag signal observed at DNMT1 OT4 can be attributed to its high sequence similarity to the on‐target site, sharing 17 identical nucleotides. Notably, while the enAsCas12a control exhibited significant off‐target cleavage as expected [28], SF01 and its variants displayed a relatively higher safety profile.

**FIGURE 7:**
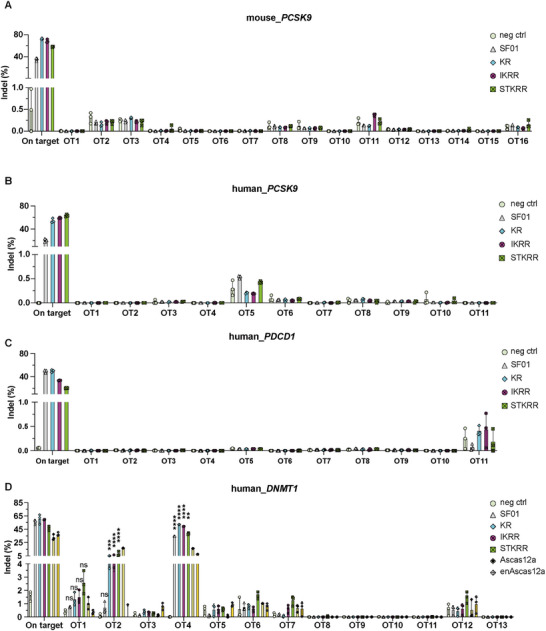
Targeted amplicon sequencing of candidate off‐target sites identified by NGS in N2a or HEK293T. Candidate off‐target sites identified by GUIDE‐tag and Digenome‐seq were analyzed by targeted amplicon deep sequencing to quantify editing frequencies in (A) mouse *PCSK9* locus, (B) human *PCSK9* locus, (C) human *PDCD1* locus, and (D) human *DNMT1* locus. Data are shown as mean ± s.d., n = 3 independent biological replicates. Statistical significance was evaluated using one‐way ANOVA followed by Dunnett's multiple comparisons test. Editing frequencies for SF01 are shown relative to the negative control (neg ctrl). Editing frequencies for KR, IKRR, and STKRR variants are presented relative to SF01. ^****^ *p* < 0.0001, ^***^ *p* < 0.001, ^**^ *p* < 0.01, ^*^ *p* < 0.05, and ns, not significant.

To determine if these candidate sites result in actual cleavages, we performed targeted deep sequencing to quantify indel formation at all candidate loci identified by both GUIDE‐tag and Digenome‐seq. Strikingly, among all sites examined, only DNMT1 OT2 exhibited off‐target editing activity that was higher than that observed (∼5%) for the parental SF01 (∼1%) (Figure 7A–D). For all other candidate off‐target sites, indel frequencies were low and statistically indistinguishable from those in negative control samples. These comprehensive results demonstrate that the substantially improved on‐target activity and broadened PAM compatibility of the SF01 variants are achieved while maintaining high genome‐wide specificity, a crucial feature for their use in therapeutic and precision research applications.

## 3 Discussion

The precise targeting of CRISPR‐Cas nucleases is fundamentally limited by their PAM requirements. In this study, we successfully engineered the compact Cas12i nuclease SF01 to create variants with dramatically expanded PAM recognition, achieving highly efficient and specific editing at near‐PAMless 5'‐NNTN‐3' sequences. Through a structure‐guided, iterative mutagenesis strategy, we identified three standout variants—KR, IKRR, and STKRR—that overcome the primary limitation of the parental enzyme. Our data suggests a defined utility for each variant: IKRR serves as a robust generalist that preserves canonical activity, while STKRR serves as a specialized tool for accessing the most challenging near‐PAMless targets.

The performance of these SF01 variants adds a powerful new tool to the expanding genome editing landscape. We recognize the significant contributions of recent protein engineering efforts that have relaxed the PAM requirements of Cas12a [7, 8, 9, 10], most notably the development of enAsCas12a and its high‐fidelity derivatives [9, 10], as well as the near‐PAMless SpRY Cas9 [19]. While these variants have undeniably broadened the targeting scope of CRISPR nucleases, our head‐to‐head comparison reveals that broad PAM recognition often necessitates a trade‐off with allele specificity. In our study, while SpRY effectively targeted the *RHO* P23H mutation, it lacked the stringency to discriminate against the wild‐type allele. In contrast, the engineered SF01 variants—specifically STKRR—demonstrated a unique capacity to access 5'‐NNTN‐3' PAMs while retaining the structural selectivity required for single‐nucleotide discrimination. This expanded targeting scope, combined with the compact size of the SF01 protein (∼1100 amino acids) relative to AsCas12a (∼1300 amino acids) and SpCas9 (∼1368 amino acids), makes these variants particularly attractive for therapeutic applications where AAV delivery and high‐fidelity editing are paramount.

Our engineering strategy, which combined AlphaFold‐based structural predictions with semi‐saturation mutagenesis, reveals a synergistic mechanism for PAM relaxation. We propose that the broad targeting capability of the STKRR and IKRR variants stems from the coupling of electrostatic compensation with structural remodeling: First, the introduction of positively charged residues (D293K, V460R, E494R) at the protein‐DNA interface likely functions as an electrostatic anchor. These residues form enhanced non‐specific salt bridges with the negatively charged DNA phosphate backbone, increasing the global DNA‐binding affinity of the nuclease. This increased affinity energetically compensates for the suboptimal interactions inherent to non‐canonical 5'‐NNTN‐3' PAM recognition, a strategy analogous to that used in previous Cas9 and Cas12a engineering efforts. Second, the T208I mutation appears to play a distinct, permissive role. Structural modeling indicates that the wild‐type threonine 208 is situated within an α‐helix of the PAM‐interacting (PI) domain, where it likely participates in a hydrogen‐bonding network that stabilizes the domain in a conformation strictly specific to the canonical PAM. We hypothesize that the substitution of the polar threonine with the hydrophobic, bulky isoleucine disrupts this “locking” network. Furthermore, the hydrophobic isoleucine side chain may pack more tightly into the hydrophobic core of the PI domain interface, stabilizing a distinct helical geometry that is more structurally distinct or flexible. This “unlocking” of the PI domain, combined with the “electrostatic glue” provided by the KR/R mutations, allows the variant to accommodate diverse PAM sequences while retaining sufficient binding energy for cleavage.

The practical utility of these variants was demonstrated across multiple applications. Their consistent high performance in different human and mouse cell lines (HEK293T, HepG2, N2a, and Hepa1‐6) underscores their broad applicability. A compelling demonstration of their therapeutic potential is the efficient and allele‐specific inactivation of the dominant‐negative *RHO* P23H mutation, a leading cause of autosomal dominant retinitis pigmentosa. This result provides a clear proof‐of‐concept for using these variants to treat genetic disorders where selective targeting of a pathogenic allele is required. Moreover, the successful development of adenine base editors (ABEs) based on the dSF01 scaffold, which achieved editing efficiencies up to 80% at endogenous loci, further broadens the utility of this platform from gene disruption to precise base correction.

While our comprehensive GUIDE‐tag and Digenome‐seq analysis indicated that the expanded targeting range of the SF01 variants does not come at the expense of specificity, we acknowledge that it may not capture all potential off‐target events in a cellular context. Future work should involve unbiased, cell‐based methods like GUIDE‐seq or DISCOVER‐seq to validate their safety profile in therapeutically relevant models [29, 30]. Additionally, while our dSF01‐ABE variants are highly active, future engineering could focus on modifying the deaminase domain to narrow the base editing window and further minimize the risk of bystander mutations [22].

Despite these promising results, we acknowledge several limitations that define the scope of future investigation. First, while we demonstrated robust allele‐specific editing of the *RHO* P23H mutation in cellular models, establishing therapeutic relevance requires validation in vivo. Future studies will focus on packaging these compact variants into AAV vectors to evaluate their delivery efficiency, safety, and therapeutic rescue in animal models of retinitis pigmentosa. Second, our current study focused on nucleases and adenine base editors (ABEs). Given the modularity of the CRISPR system, the engineered SF01 variants offer a versatile chassis for further expansion. Their compact size (∼1100 aa) provides a distinct advantage for constructing larger fusion systems that are often size‐restricted, such as cytidine base editors (CBEs), prime editors (PEs), or epigenetic editors (EEs). Adapting the KR and IKRR variants into these frameworks could significantly broaden the scope of precise genomic corrections accessible for human gene therapy.

In conclusion, the engineered SF01 variants KR, IKRR, and STKRR constitute a significant expansion of the CRISPR genome editing toolkit. By offering near‐PAMless targeting with high efficiency and specificity in a compact package, these nucleases overcome a major bottleneck in the field and open the door to new research and clinical applications that were previously out of reach.

## 4 Materials and Methods

### 4.1 Plasmid Cloning

Plasmids were constructed using standard molecular cloning techniques, including PCR amplification, restriction enzyme digestion, T4 DNA ligation, and Gibson assembly. All PCR amplifications were carried out using FastPfu Fly DNA Polymerase (TransGen Biotech, AP231‐13) according to the manufacturer's instructions. A synthetic single‐mutant library was generated by site‐directed mutagenesis using mutagenic primers, and the resulting variants were assembled into expression vectors via Gibson Assembly (ClonExpress Ultra One Step Cloning Kit, Vazyme, C115‐02). Multiple‐mutation variants were subsequently constructed based on selected single mutants using the same mutagenesis strategy. crRNA oligonucleotides were synthesized by Beijing Tsingke Biotech, annealed, and subsequently ligated into the BbsI (New England BioLabs) restriction site of the all‐in‐one expression vector. dSF01 variants were generated by introducing single mutations at four nuclease‐active‐site residues (D619A, N621A, E844A, and D1017A), as well as all pairwise combinations of these mutations, yielding a total of ten dSF01 constructs. The IVT vectors for SF01 and its variants (KR, IKRR, STKRR) were constructed by inserting their coding sequences into the PEmax mRNA plasmid backbone (Addgene, #204472) that had been digested with SalI and EcoRI, using Gibson assembly. Ligated products were transformed into DH5α *E. coli* cells. A complete list of oligonucleotide sequences used in this study is provided in Table S2. The final constructed vectors were all validated for accuracy through Sanger sequencing.

### 4.2 Protein Expression and Purification

Protein expression and purification [28, 31] for SF01 and its variants (KR, IKRR, STKRR), AsCas12a, and enAsCas12a were carried out using pET‐21a protein expression plasmids (Novagen) [9]. The plasmid encoding each Cas12 protein was transformed into *E. coli* Rosetta 2 (DE3) pLysS Chemically Competent Cells (WEIDI, EC1016) for protein overexpression. Cells were cultured at 37°C until the OD_600_ reached 0.6–0.8, followed by a 1‐h incubation on ice. Protein expression was then induced by adding IPTG to a final concentration of 1 mm, and the cells were further incubated at 18°C for 16 h.

After induction, cells were harvested by centrifugation and resuspended in Ni‐NTA binding buffer (20 mm TRIS, 1 m NaCl, 20 mm imidazole, 1 mm TCEP, pH 7.5), supplemented with Protease Inhibitor Cocktail (MCE, HY‐K0010). The cell suspension was lysed using a high‐pressure homogenizer (ATSHPH, AH‐BASIC 30) according to the manufacturer's protocol.

For protein purification, the lysate was first passed through Q Agarose 6FF (YEASEN, 20547ES25), and the flow‐through was collected. The flow‐through crude protein was then applied to Ni‐NTA resin, washed with binding buffer, and proteins were eluted using an elution buffer containing 20 mm TRIS, 500 mm NaCl, 250 mm imidazole, and 10% glycerol (pH 7.5). The purified Cas12 proteins were concentrated using a 50 kDa centrifugal filter (Millipore) and exchanged into SEC buffer (20 mm HEPES, 500 mm NaCl, 1 mm EDTA, 10% glycerol, pH 7.5) at 4°C. The concentrated protein was further purified by application to Size‐exclusion chromatography (SEC) using a Superdex 200 Increase 10/300 GL column (Cytiva, 28990944). The Cas12 protein peak was collected, concentrated again using a 50 kDa filter (Millipore), and assessed for purity. The purity of the final protein was confirmed to be >95% by WinnerPage Protein Gel (CWBIO, CW8209S), and protein concentration was quantified using the Easy Protein Quantitative Kit (Bradford) (Transgen, DQ101‐01).

### 4.3 DNA Cleavage Assays

For the enzyme activity validation of the purified proteins, a 744 bp substrate containing the *DNMT1* target was amplified from HEK293T genomic DNA via PCR. The *DNMT1* crRNA was chemically synthesized by GenScript, with sequence details provided in Table S2. The cleavage assay was performed in a reaction mixture containing 30 nM protein, 50 nm crRNA, and 500 ng PCR product in rCutSmart Buffer (NEB, B6004S). The protein and crRNA were incubated at 37°C for 10 min to form the RNA‐protein complex, followed by the addition of the PCR product. After 30 min of incubation at 37°C, the reaction was terminated by adding 1 µL RNase A (TransGen, GE101‐01) and 1 µL Proteinase K (TransGen, GE201‐01), followed by an additional 10‐min incubation at 37°C to degrade the RNP complex. The reaction mixture was then purified using the DNA Clean & Concentrator‐5 kit (Zymo, D4013) and analyzed by 1.5% agarose gel electrophoresis.

For the kinetic experiments, the 5′‐FAM‐labeled nontarget strand, containing different PAM sequences (ATTA CATG CCTC AGTG), was synthesized by GenScript. The complementary ssDNA was synthesized by Sangon Biotech, with the sequence provided in Table S2. The 5′‐FAM‐labeled nontarget strand was annealed with its complementary target strand to form a double‐stranded DNA (dsDNA). Reactions were carried out with 30 nm protein, 50 nM crRNA, and 5 nM 5′‐FAM‐labeled dsDNA. The reactions were quenched at specified time points using 2× TBE‐Urea loading buffer (Sangon Biotech, C506046‐0005). After denaturation at 95°C for 3 min, the cleavage products were separated by 15% urea–PAGE and visualized using blue light imaging (Tanon, MINI Space). The in vitro cleavage percentage was calculated using the following formula:

$$\begin{array}{cc} & \mathrm{cleavage}\%=\mathrm{Intensity}\;\mathrm{of}\;\mathrm{cleaved}\;\mathrm{product}/\\ & \;(\mathrm{Intensity}\;\mathrm{of}\;\mathrm{cleaved}\;\mathrm{and}\;\mathrm{uncleaved}\;\mathrm{products})\;\times \;100\% \end{array}$$

### 4.4 RNA Production and Purification

The crRNAs required for in vitro experiments were chemically synthesized by GenScript, with sequences listed in Table S2. mRNAs encoding SF01 and its variants (KR, IKRR, STKRR) were transcribed in vitro (IVT). Plasmid DNA templates were first linearized using the PmeI restriction enzyme, which cleaves downstream of the poly(A) tail sequence. A total of 500 ng of linearized DNA was then used as the template for the transcription reaction. The reaction mixture contained T7 RNA polymerase (Novoprotein, GMP‐E121), inorganic pyrophosphatase (Novoprotein, GMP‐M036), RNase inhibitor (Novoprotein, GMP‐E125), and a mixture of nucleoside triphosphates (ATP, GTP, CTP). For co‐transcriptional capping, CleanCap AG (TriLink Biotechnologies) was included, and N1‐methylpseudouridine‐5′‐triphosphate (m1ΨTP, TriLink Biotechnologies) was substituted for UTP to reduce immunogenicity and enhance translation efficiency. After incubation at 27°C for 2 h, the DNA template was removed by treatment with DNase I, and the resulting mRNA was purified using LiCl precipitation (7.5 m) (Invitrogen, AM9480). The purified mRNA was then eluted in nuclease‐free water. mRNA concentration was determined using a NanoDrop One spectrophotometer (Thermo Fisher Scientific), and its integrity was assessed by electrophoresis on a 1% MOPS‐agarose gel. The purified mRNA was stored at −80°C for subsequent use.

### 4.5 Cell Culture and Transfection

HEK293T (RRID: CVCL_0063), N2a (Neuro‐2a mouse neuroblastoma cells, RRID: CVCL_0470), Hepa1‐6 (mouse Hepatoma cells, RRID: CVCL_0327), and HepG2 (Human Hepatocellular Carcinoma, CVCL_0027) cells were obtained from the Cell Bank of the Chinese Academy of Sciences and confirmed to be mycoplasma‐free by Mycolor One‐Step Mycoplasma Detector (Vazyme, D201‐01). HEK293T, N2a, Hepa1‐6, and HepG2 were cultured in DMEM (Gibco, C11995500bt) supplemented with 10% (v/v) fetal bovine serum (Gibco, 10099141) and 1% penicillin/streptomycin (Gibco, 15140122) at 37°C with 5% CO_2_. All experiments were performed using cells between passages 5 and 20 after thawing. To ensure optimal proliferation, the culture medium for cells was refreshed every 48 h. Cells were passaged using 0.25% trypsin‐EDTA solution (Gibco, 25200056) when they reached 80%–90% confluence.

HEK293T cells were maintained in standard culture conditions and seeded into 12‐well plates at approximately 80% confluency prior to transfection. For each transfection, 1 µg of plasmid DNA was diluted in 100 µL of Opti‐MEM (Gibco, 51985091), followed by the addition of 3 µL polyethyleneimine (PEI, Yeasen, 40816ES02). The DNA/PEI complexes were incubated for 20 min at room temperature before being added to the cells. For the EGxxFP reporter assay, cells were harvested 48 h post‐transfection and analyzed by flow cytometry. For endogenous target validation, cells were subjected to puromycin (Thermo Fisher, A1113803) selection (2 µg/mL) for 5 days prior to collection and downstream analysis. To assess ABE editing activity, 150 000 mCherry‐positive cells were sorted 4 days after transfection, followed by genomic DNA extraction and sequencing. HepG2, Hepa1‐6, and N2a cells were transfected using the OPTIMUS DNA Transfection Reagent (Polyplus, 101000006) following the manufacturer's standard protocol.

For mRNA delivery, Hepa1‐6 cells were electroporated using the Neon Electroporation System (Thermo Fisher Scientific, 10 µL kit) with optimized parameters specific to the cell type (1,350 V, 20 ms, 2 pulses). In each electroporation reaction, 6 × 10^4^ cells were harvested by centrifugation (300 × g for 5 min), resuspended in 10 µL of Neon Buffer R, and mixed with 3 µg of mRNA, 80 pmol of the corresponding gRNAs, and 3 µL of RNase inhibitor. After electroporation, genomic DNA was extracted 72 h post‐transfection and stored at −80°C for subsequent library preparation and targeted amplicon deep sequencing. RNA was isolated for further use in RT‐qPCR (quantitative real‐time PCR) analysis.

To generate a stable HEK293T cell line expressing both *RHO* and *RHO* P23H, the CMV‐RHO‐T2A‐copGFP and SFFV‐RHO‐P23H‐P2A‐mTagBFP2 expression cassettes were synthesized by GeneScript and integrated into a piggyBac plasmid‐based modular vector via Gateway cloning technology. This vector was then co‐transfected into HEK293T cells with CMV‐super piggyBac transposase at a 5:1 ratio, using PEI (Polyethylenimine). After 48 h, cells were sorted based on dual fluorescence (copGFP and mTagBFP2), and double‐positive clones were isolated for further analysis.

### 4.6 RNA Isolation and RT‐qPCR

Total RNA was isolated from cells using the RNA Easy Fast Animal Tissue/Cell Total RNA Extraction Kit (TIANGEN, DP451). cDNA synthesis was carried out using the HiScript IV All‐in‐One Ultra RT SuperMix for qPCR (Vazyme, R433). Quantitative PCR (qPCR) was performed with SupRealQ Ultra Hunter SYBR qPCR Master Mix (U+) (Vazyme, Q713) on a QuantStudio 5 Real‐Time PCR System (Thermo Fisher Scientific). Gene expression levels of mouse *PCSK9* and *GAPDH* (as a housekeeping control) were measured using specific primers. Relative fold changes (FCs) in gene expression were determined using the ΔΔCT method [32].

### 4.7 Flow Cytometry

Transfected cells of each well were digested with 300 µL 0.25% trypsin (Gibco, 25200‐056) after removing the medium. Then, 300 µL of medium was added to terminate the digestion, and the cells were resuspended in 500 µL of medium after 3 min of centrifugation at 1000 rpm. Cells were then pipetted into a 5‐mL round‐bottom polystyrene test tube with a cell strainer snap cap (Corning, 352235). Flow cytometry data were collected using a CytoFLEX (Beckman Coulter Life Sciences) and analyzed using CyExpert version 2.5.0.77 software. For the assay of ABE endogenous targets, cells were sorted using BD FACSAria III (Becton Dickinson and Company).

Scatter gates were applied to remove nonviable cells and doublets. For positive experiments, gates were applied based on cells transfected with both mCherry and GFP signals. mCherry fluorescence was detected using Y610 (561/610 nm). GFP fluorescence was detected using B525 (488/525 nm). Approximately 30 000 cells (after scatter gating) were collected for each sample. The editing efficiency was calculated as the number of GFP^+^ cells divided by the number of mCherry^+^ cells.

### 4.8 Next‐Generation Sequencing

Deep sequencing was performed to assess the genome editing efficiency of Cas nucleases or ABE at endogenous loci. HEK293T, N2a, and HepG2 cells were harvested and lysed by resuspension in DNAiso Reagent (Takara, 9770A) at room temperature for 5 min. Genomic DNA was then extracted using DNA Selection Beads (Yeasen, 12601ES08) according to the manufacturer's protocol and eluted in nuclease‐free water. Target loci were amplified from the purified genomic DNA using Phanta Max Super‐Fidelity DNA Polymerase (Vazyme, Cat. No. P505). The amplicons were subjected to next‐generation sequencing (NGS) on the DNBSEQ‐G99 platform (MGI Tech, G99). Endogenous target sequences used are listed in Table S2. Sequencing data were analyzed using the *CRISPResso2* [33]. Indel rates and base editing outcomes were assessed through two distinct computational pipelines:

CRISPResso –fastq_r1 READS1_DATA –fastq_r2 READS2_DATA –amplicon_seq REFERENCE_SEQUENCE ‐guide_seq GUIDE_SEQUENCE ‐q 30 ‐wc 1 ‐w 20 –plot_window_size 24 ‐o OUTPUT_FILE CRISPResso –fastq_r1 READS1_DATA –fastq_r2 READS2_DATA –amplicon_seq REFERENCE_SEQUENCE ‐guide_seq GUIDE_SEQUENCE ‐q 30 ‐wc ‐17 ‐w 20 –plot_window_size 24 ‐o OUTPUT_FILE –base_editor_output. In these commands, –fastq_r1 and –fastq_r2 refer to the pair‐end sequencing results after barcode splitting. The –amplicon_seq indicates the complete amplified sequence region, while the ‐guide_seq is the guide RNA sequence immediately adjacent to but not including the PAM sequence. Parameter ‐q specifies the phred33 quality threshold, retaining only reads meeting the minimum average quality score requirement. The ‐wc and ‐w parameters configure the quantification window's central position and dimensional scale relative to the guide RNA 3’ terminus, whereas ‐plot_window_size modulates the visualization scope for indel patterns. During base editing analysis, activation of –base_editor_output enables CRISPResso2 to catalog A•G substitutions within the specified region. This integrated methodology facilitates comprehensive evaluation of targeted locus modifications, encompassing both indel induction frequencies and base conversion efficiencies.

### 4.9 Immunofluorescence and Flow Cytometry Analysis of Cell Surface Marker Expression

Following transfection, cells were assessed for the expression of the characteristic bMSC surface marker CD29 (BioLegend, 303008) via immunofluorescence staining and flow cytometry. Cells were washed three times with ice‐cold PBS and incubated with the APC‐conjugated anti‐CD29 antibody (R660/10 nm) for 15 min at 4°C. After staining, unbound antibodies were removed by washing with PBS. During flow cytometry, scatter gating was applied to eliminate nonviable cells and doublets. Positive populations were defined using gating parameters established from cells stained with the APC fluorophore.

### 4.10 Analysis of PAM Recognition Profile of SF01

To systematically evaluate the PAM recognition profile of SF01, we modified the PAM sequence of the T8 locus within the EGxxFP reporter system to generate all possible 5′‐NVTN‐3′ PAM variants (V = A/C/G). This comprehensive panel was used to assess SF01 activity across diverse PAM contexts in mammalian cells.

### 4.11 GUIDE‐Tag Assay for Off‐Target Analysis

The genome‐wide off‐target activity of SF01 and its engineered variants was assessed using the GUIDE‐tag method [24, 25], as previously described, with minor modifications. Briefly, HEK293T cells (500 000) were electroporated (Neon NxT, Thermo Fisher Scientific) with 1 µg of an all‐in‐one plasmid encoding SF01 and the corresponding crRNA, along with 10 pmol of annealed GUIDE‐tag oligonucleotides. Forty‐eight hours post‐transfection, genomic DNA was extracted for downstream analysis.

### 4.12 Digenome‐Seq Assay for Off‐Target Analysis

The Digenome‐seq assay was conducted following a standard protocol with minor modifications [27]. High‐quality genomic DNA (gDNA) was isolated from HEK293T and N2a cells using the DNeasy Blood & Tissue Kit (Qiagen, 69504). Ribonucleoprotein (RNP) complexes were assembled according to the DNA cleavage assay protocol. The mouse *PCSK9*, human *PDCD1*, and human *PCSK9* crRNA sequences (provided in Table S2) were synthesized by GenScript. For the digestion reaction, a mixture containing 50 nm protein, 100 nm crRNA, and 4 µg gDNA in rCutSmart Buffer (NEB, B6004S) was incubated at 37°C for 8 h. Four independent reactions were carried out for each sample to ensure adequate input for downstream analysis. The reaction was subsequently terminated by the addition of 2.5 µL RNase A (TransGen, GE101‐01), followed by a 30‐min incubation at 37°C. Afterward, 2.5 µL Proteinase K (TransGen, GE201‐01) was added, and the mixture was incubated for an additional 30 min at 37°C to digest the RNP complex. The digested DNA was purified using the DNA Clean & Concentrator‐25 Kit (Zymo, D4033). The purified gDNA was then fragmented using a Bioruptor Plus (Diagenode) and prepared for library construction with the TransNGS DNA Library Prep Kit for Illumina (TransGen, KP201‐11‐V2). The resulting DNA libraries were subjected to next‐generation sequencing (NGS) on the DNBSEQ‐G99 platform (MGI Tech, G99) for comprehensive off‐target analysis.

### 4.13 Statistical Analysis

All statistical analyses were performed using GraphPad Prism 10.1.0 (GraphPad Software). Unless otherwise specified, data are presented as mean ± standard deviation (s.d.). Statistical tests were chosen according to the experimental design as follows: For comparisons between two groups, two‐tailed unpaired Student's t‐tests were used. For comparisons involving three or more groups (single factor), one‐way ANOVA followed by Dunnett's multiple comparisons test was applied. For multi‐target or multi‐factor experiments (e.g., assessments across targets), two‐way ANOVA was used to compare all group means. Statistical significance thresholds used in figures are indicated by asterisks: ^****^ *p* < 0.0001, ^***^ *p* < 0.001, ^**^ *p* < 0.01, ^*^ *p* < 0.05, and ns, not significant. No data were excluded from the analyses unless explicitly stated, and all experiments were independently repeated at least three times to ensure reproducibility.

## Author Contributions

Q.C., H.G., Y.L., P.L., and J.‐K.Z. conceptualized the project. Q.C., H.G., Y.L., and P.L. designed the experiments. Q.C. and H.G. conducted molecular biological and cellular experiments. Q.C., H.G., and P.L. conducted high‐throughput sequencing and bioinformatics analyses. Q.C., H.G., Y.L., P.L., and J.‐K.Z. interpreted the data and wrote the paper, and all authors edited the paper.

## Conflicts of Interest

Southern University of Science and Technology has filed a patent application related to this work with some co‐authors listed as inventors.

## Supporting information




**Supporting File**: advs73816‐sup‐0001‐SuppMat.docx.

## Data Availability

The data that support the findings of this study are openly available in Sequence Read Archive at https://www.ncbi.nlm.nih.gov/bioproject/PRJNA1307282, reference number 1307282.
