# Supplementary material for: Structure‐Guided Engineering of a Cas12i Nuclease Unlocks Near‐PAMless Genome Editing
Source: Adv Sci (Weinh). 2026 Jan 14;13(17):e16670. doi: 10.1002/advs.202516670 (PMC13042403; doi:10.1002/advs.202516670)
Supplement: Supplementary file 1 — Supporting File: advs73816‐sup‐0001‐SuppMat.docx. [file ADVS-13-e16670-s001.docx]

**Supporting Information**

**Structure-guided Engineering of a Cas12i Nuclease Unlocks Near-PAMless Genome Editing**

Author: Qitong Chen^1,3^, Hanlin Gou^1,3^, Chao Xu^1^, Sihan Wang^1^, Huitao Zhang^1^, Minglei Song^1^, Mengge Wang^1^, Xingkun Ji^1^, Xiaofei Wei^1^, Yuanyan Tan^1^, Hehua Quan^1^, Pengyu Luo^1^, Hanyu Shou^2^, Pengpeng Liu^1,*^, Yafeng Liang^1,*^, Jiankang Zhu^1,*^

^1^Institute of Advanced Biotechnology and School of Medicine, Southern University of Science and Technology, Shenzhen 518055, China

^2^Zhejiang University–University of Edinburgh Institute, Zhejiang University, Hangzhou, 310003, China

^3^These authors contributed equally

12231423@mail.sustech.edu.cn (Q. Chen), 12531566@mail.sustech.edu.cn (H. Gou), 12231426@mail.sustech.edu.cn (C. Xu), 18081714d@connect.polyu.hk (S. Wang), HuitaoZhang0104@163.com (H. Zhang), songml@sustech.edu.cn (M. Song), 12231425@mail.sustech.edu.cn (M. Wang), 12331369@mail.sustech.edu.cn (X. Ji), 12231424@mail.sustech.edu.cn (X. Wei), tanyy3@mail.sustech.edu.cn (Y. Tan), 12431384@mail.sustech.edu.cn (H. Quan), 12231422@mail.sustech.edu.cn (P. Luo), hanyu.24@intl.zju.edu.cn (H. Shou)

^*^Correspondence to: [liupp@sustech.edu.cn](mailto:liupp@sustech.edu.cn) (P. Liu), [liangyf@mail.sustech.edu.cn](mailto:liangyf@mail.sustech.edu.cn) (Y. Liang), [zhujk@sustech.edu.cn](mailto:zhujk@sustech.edu.cn) (J. Zhu)


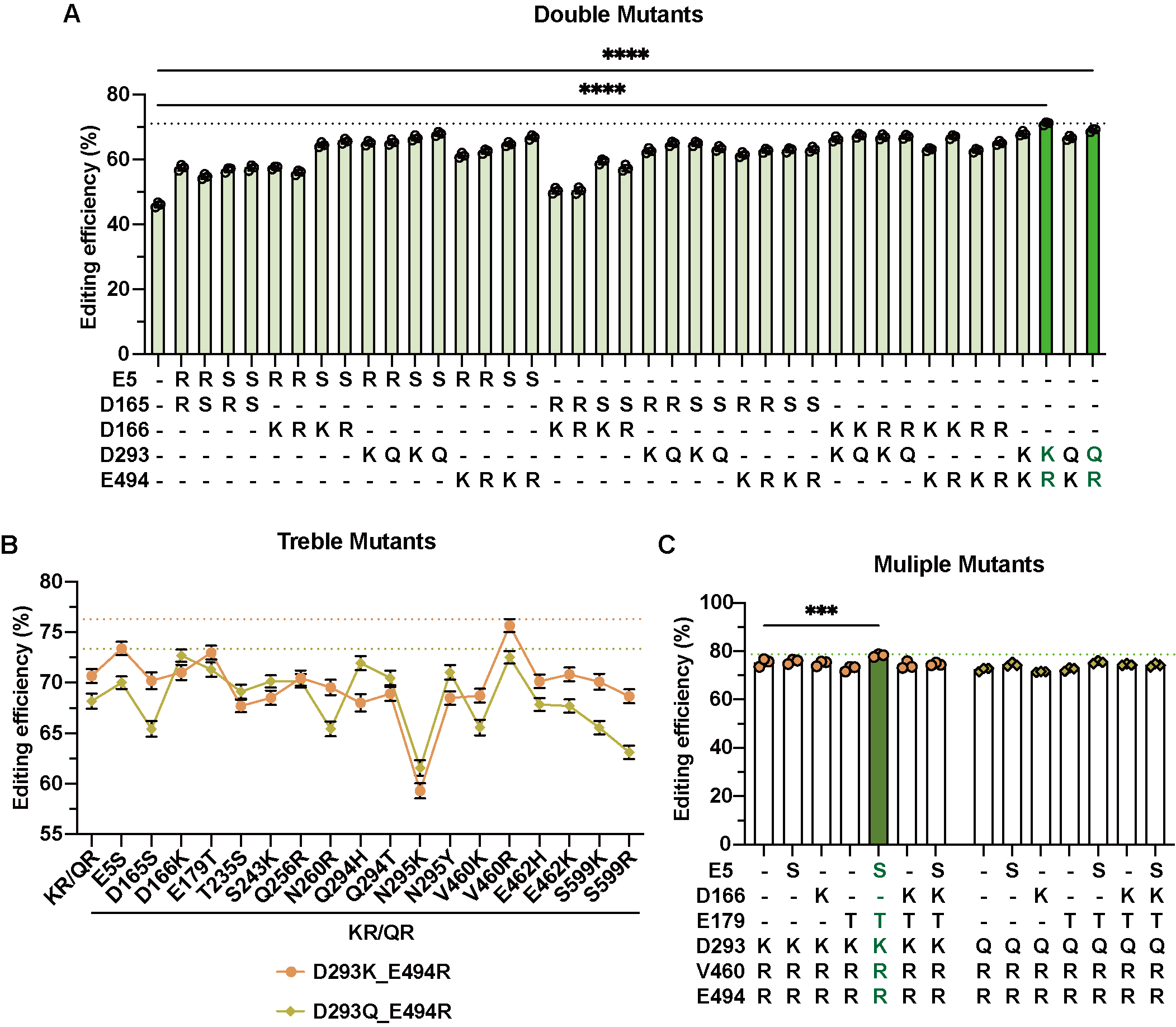


**Figure S1. Stepwise combinatorial engineering of SF01 variants in HEK293T cells.**

(A) Pairwise mutagenesis of five key residues (E5, D165, D166, D293, E494) yielded 40 double mutants. KR (D293K_E494R) and QR (D293Q_E494R), highlighted in green, were identified as the top-performing combinations among all double mutants. Dashed line indicates the editing efficiency of KR. Data are shown as mean ± s.d., n = 3 independent biological replicates.

(B) Additional substitutions were introduced into KR and QR to generate triple mutants. Orange and yellow dashed lines represent the highest-performing variants KRR and QRR, respectively. Data are shown as mean ± s.d., n = 3 independent biological replicates.

(C) Higher-order variants (≥4 mutations) were generated, with STKRR (highlighted in green) showing superior activity. Dashed line indicates its editing efficiency. Data are shown as mean ± s.d., n = 3 independent biological replicates.

Statistical significance was evaluated using one-way ANOVA followed by Dunnett's multiple comparisons test. *****p* < 0.0001, ****p* < 0.001, ***p* < 0.01, **p* < 0.05, and ns, not significant.


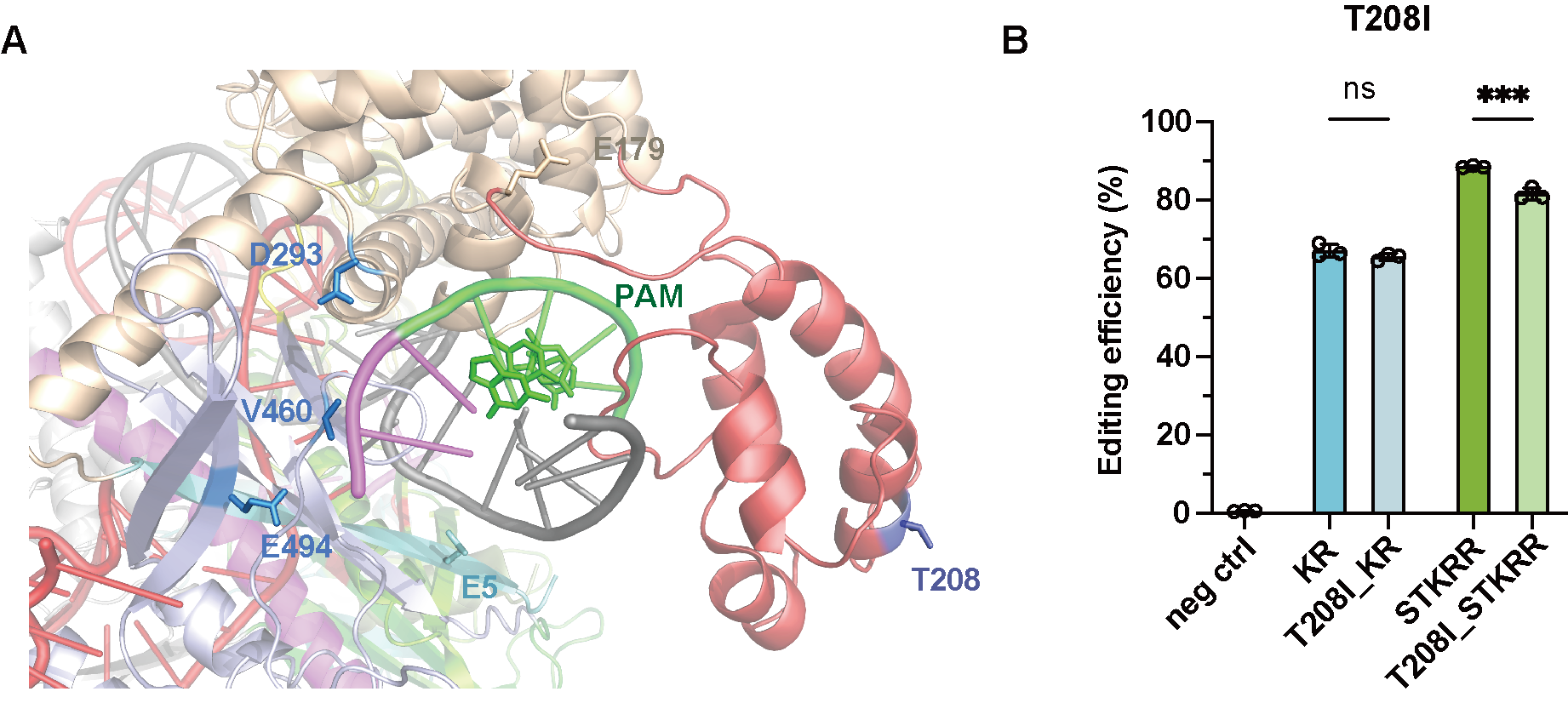


**Figure S2. Identification and functional characterization of the T208I substitution.**

(A) Structural localization of T208 (blue) within the PI domain (red). The PAM is highlighted in green. Mutation sites constituting the STKRR variant are indicated.

(B) Editing efficiencies of KR and STKRR variants with the T208I substitution in HEK293T cells. Data are shown as mean ± s.d., n = 3 independent biological replicates.

Statistical significance was evaluated using one-way ANOVA followed by Dunnett's multiple comparisons test. *****p* < 0.0001, ****p* < 0.001, ***p* < 0.01, **p* < 0.05, and ns, not significant.


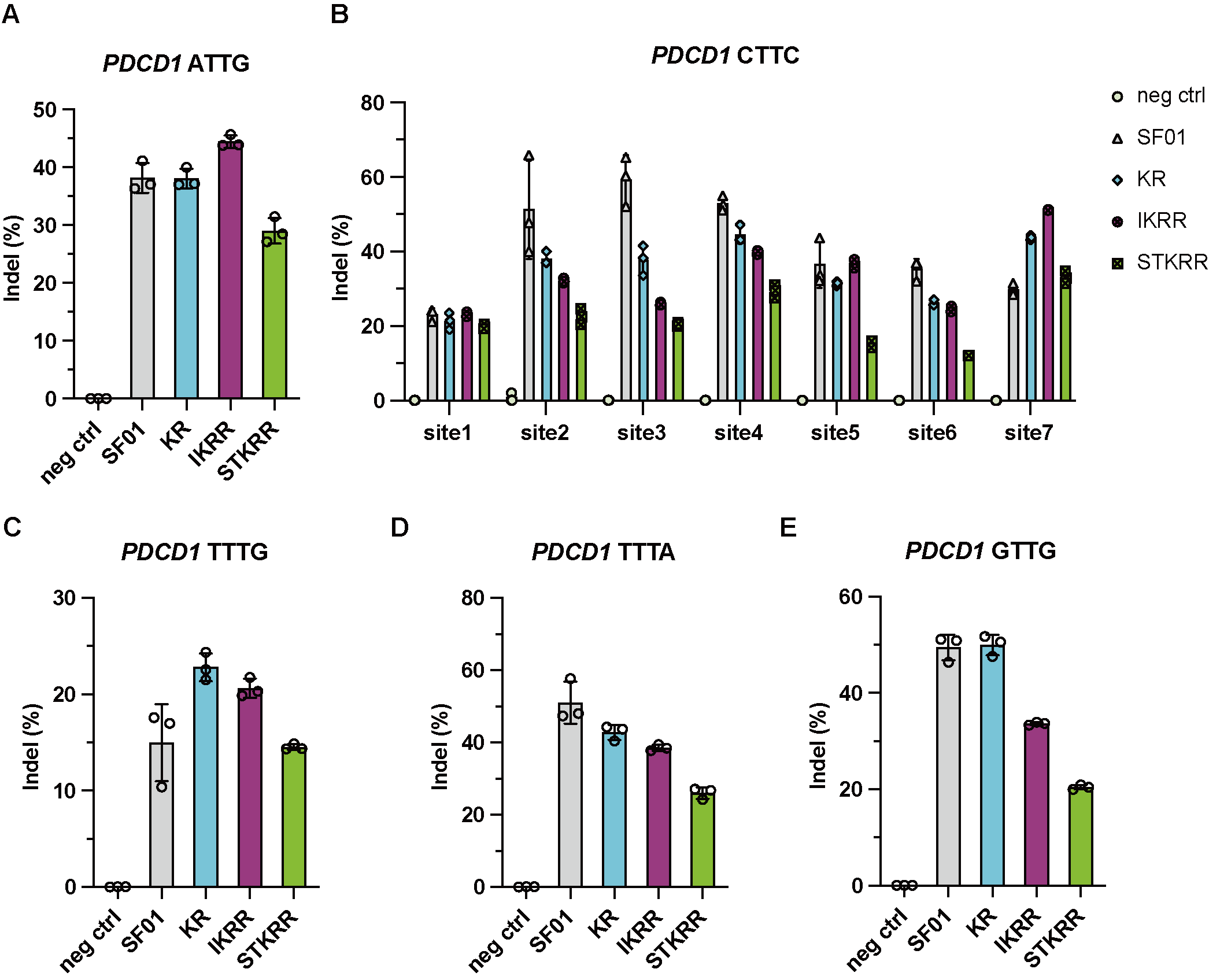


**Figure S3. Editing efficiencies of SF01 and its variants (KR, KRR, STKRR) at 11 endogenous target sites within the *PDCD1* gene bearing canonical NTTN PAMs in HEK293T cells.**

(A–E) Indel frequencies by NGS at *PDCD1* sites with ATTG (A), CTTC (B), TTTG (C), TTTA (D), and GTTG (E) PAM. Data are shown as mean ± s.d., n = 3 independent biological replicates.


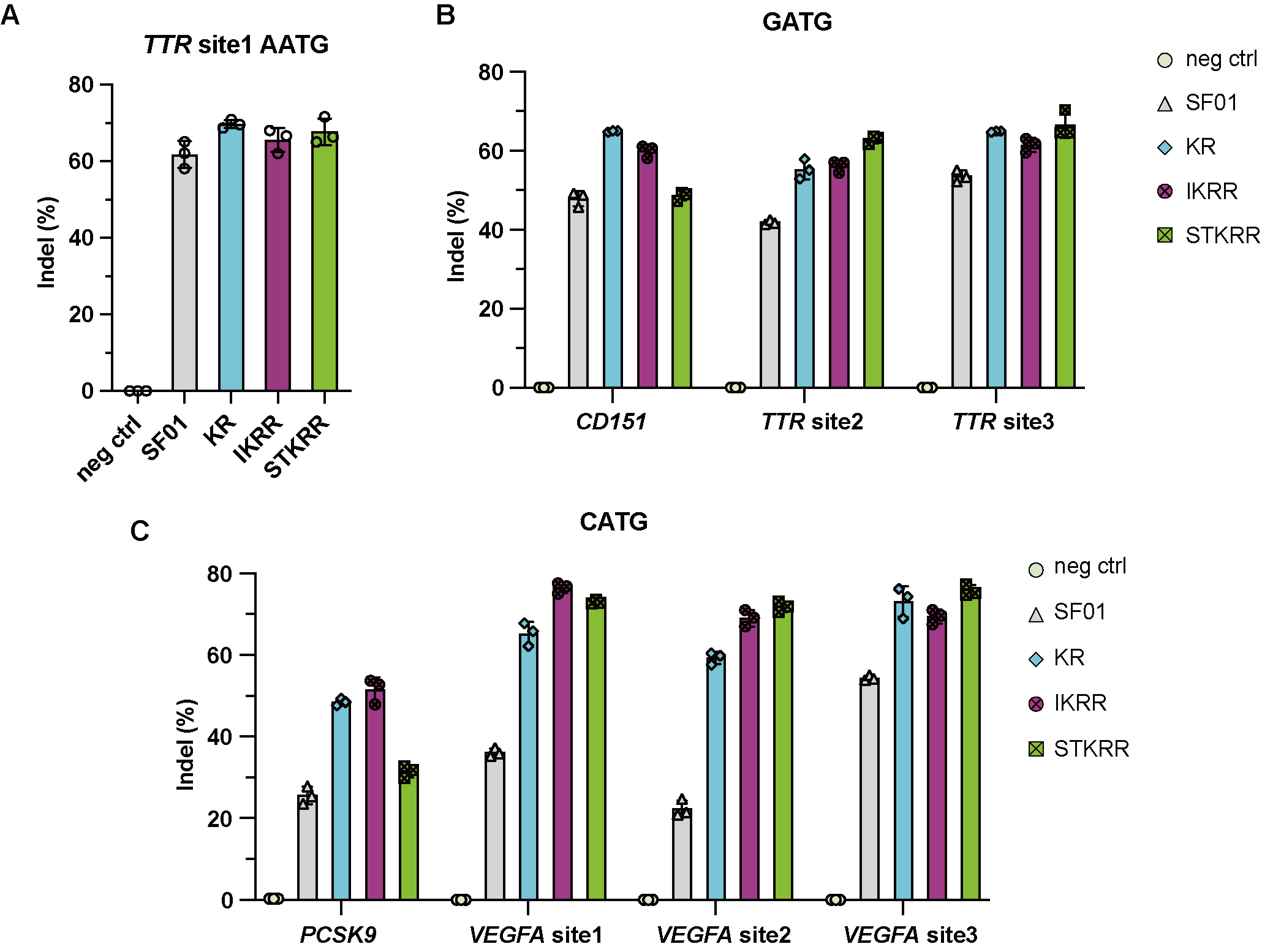


**Figure S4. Editing efficiencies of SF01 and its variants (KR, KRR, STKRR) at endogenous NATG PAM sites within *CD151*, *PCSK9*, *TTR*, and *VEGFA* genes in HEK293T cells.**

(A–C) Indel frequencies by NGS at *TTR* site1 with AATG PAM (A), *CD151* and *TTR* with GATG PAM (B), *PCSK9* and *VEGFA* with CATG PAM (C). Data are shown as mean ± s.d., n = 3 independent biological replicates.


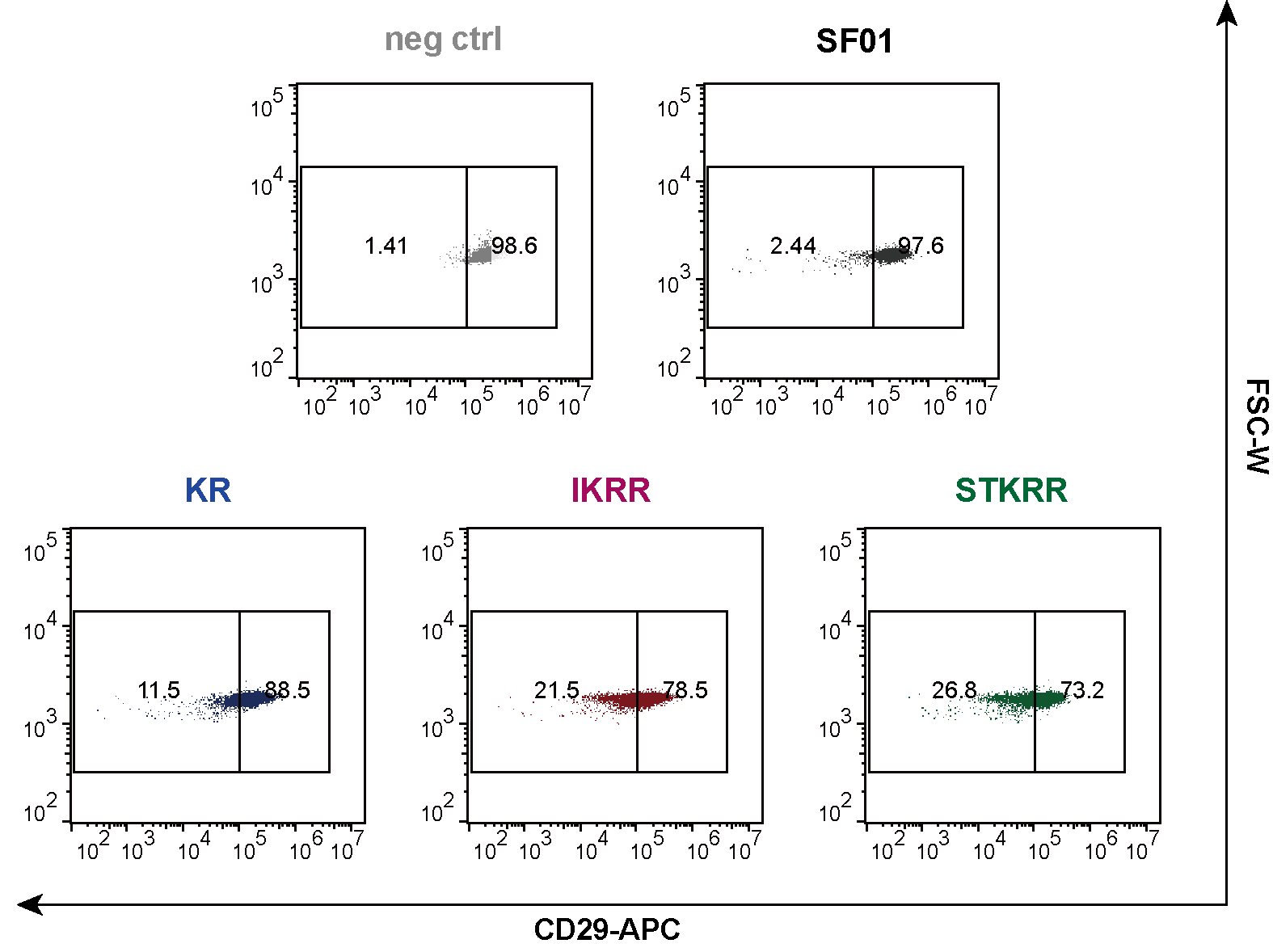


**Figure S5. Flow cytometry analysis of CD29 expression after editing by SF01 and its variants (KR, KRR, STKRR).**

Representative flow cytometry plots showing CD29-APC fluorescence intensity (x-axis) versus forward scatter width (FSC-W, y-axis) in HEK293T cells.

**
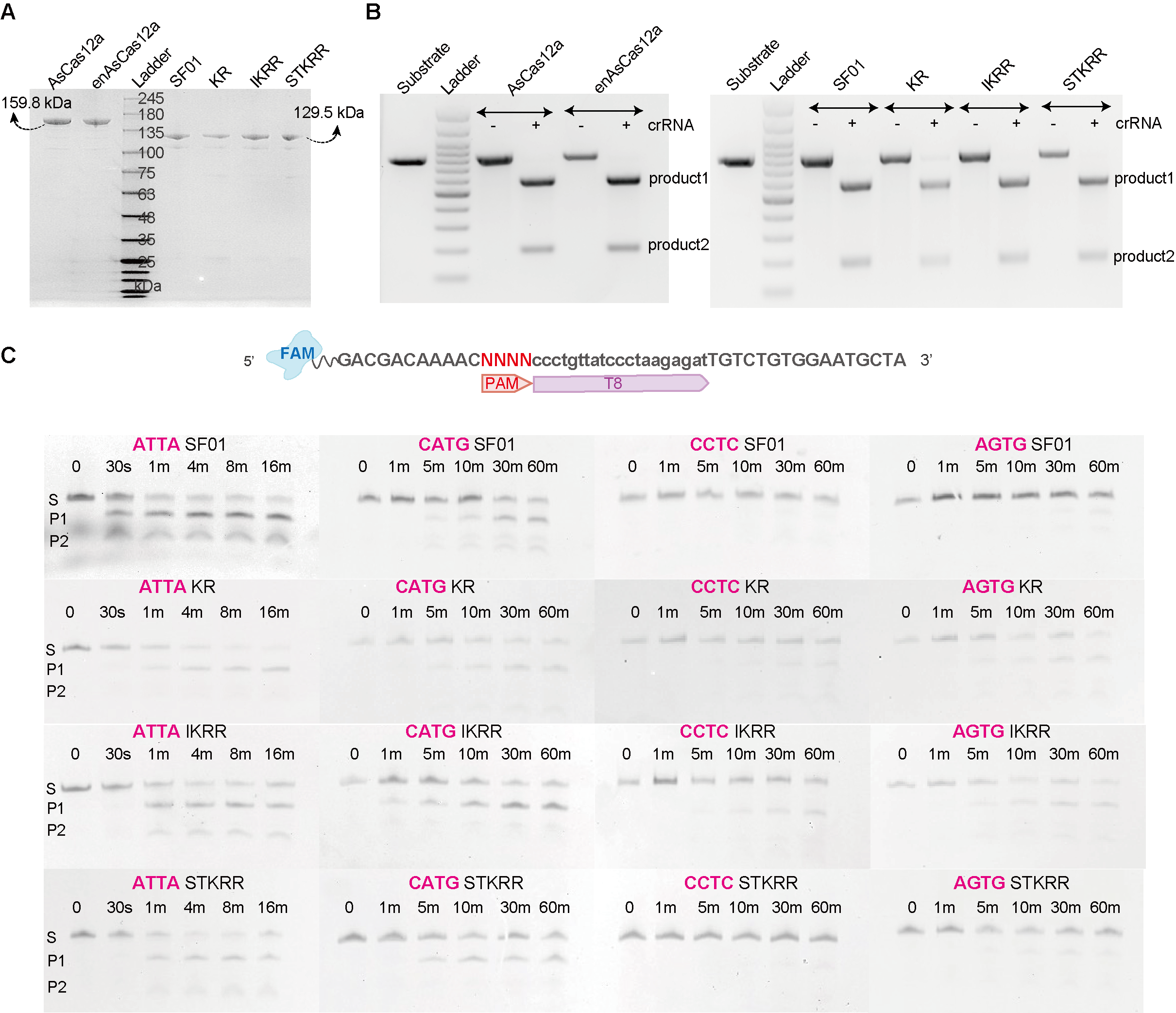
**

**Figure S6. *In vitro* kinetic evaluation of SF01, KR, IKRR, and STKRR.**

(A) An SDS-PAGE gel image of the purified AsCas12a, enAsCas12a, SF01, KR, IKRR, and STKRR.

(B) *In vitro* nuclease activity of AsCas12a, enAsCas12a, SF01, KR, IKRR, and STKRR using a *DNMT1* targeting substrate.

(C) *In vitro* cleavage assays with various synthetic DNA substrates. The upper panel shows the schematic design of the DNA substrates, which were labeled with a 5′ FAM and incorporated different PAM motifs. Reaction products were resolved on 15% urea–PAGE gels and imaged in the lower panel.

**
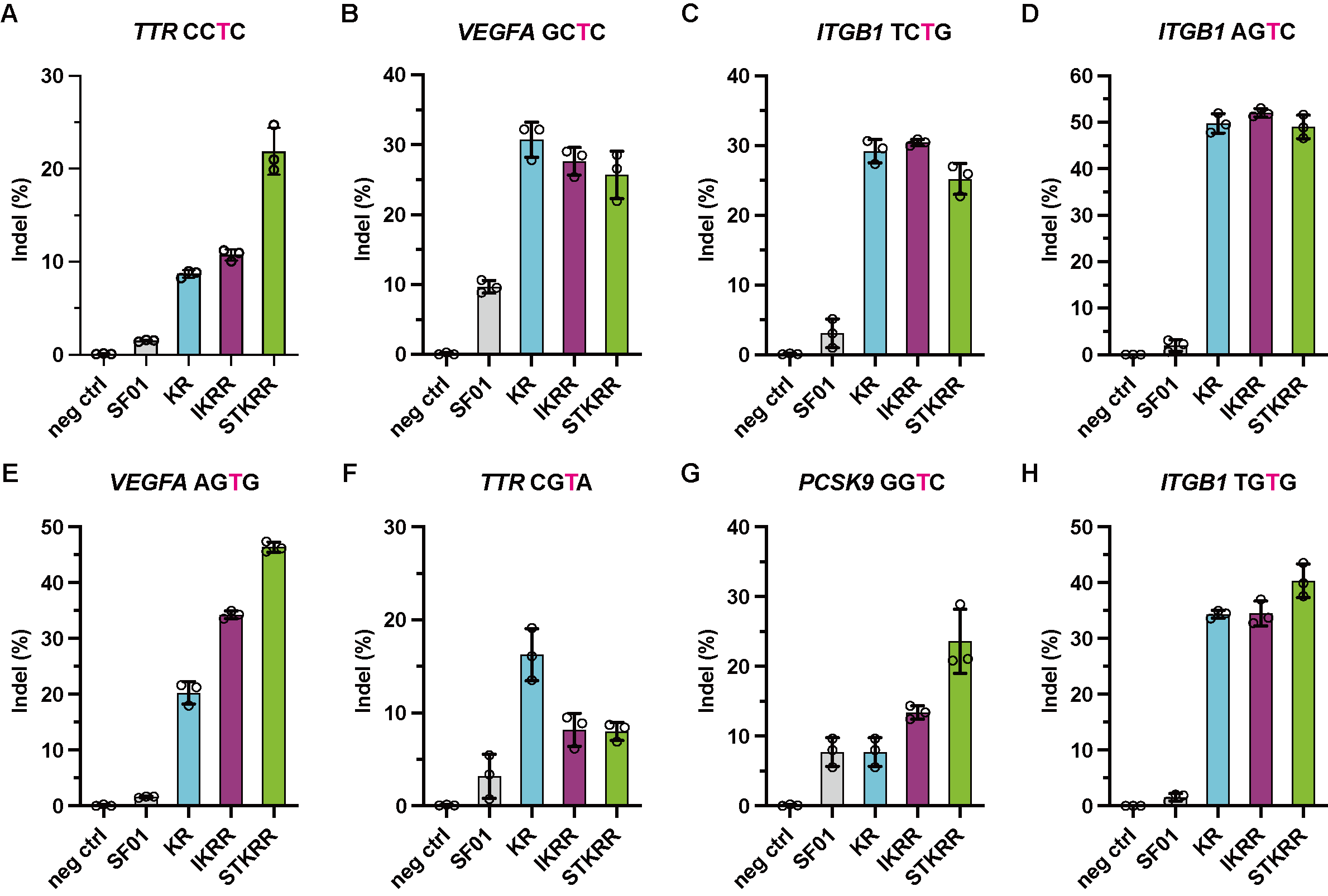
**

**Figure S7. Editing efficiencies of SF01 and its variants (KR, KRR, STKRR) at endogenous NSTN PAM within *ITGB1*, *PCSK9*, *TTR*, and *VEGFA* genes in HEK293T cells.**

(A–H) Indel frequencies by NGS at *TTR* with CCTC PAM (A), *VEGFA* with GCTC PAM (B), *ITGB1* with TCTG PAM (C), *ITGB1* with AGTC PAM (D), *VEGFA* with AGTG PAM (E), *TTR* with CGTA PAM (F), *PCSK9* with GGTC PAM (G), and *ITGB1* with TGTG PAM (H). Data are shown as mean ± s.d., n = 3 independent biological replicates.


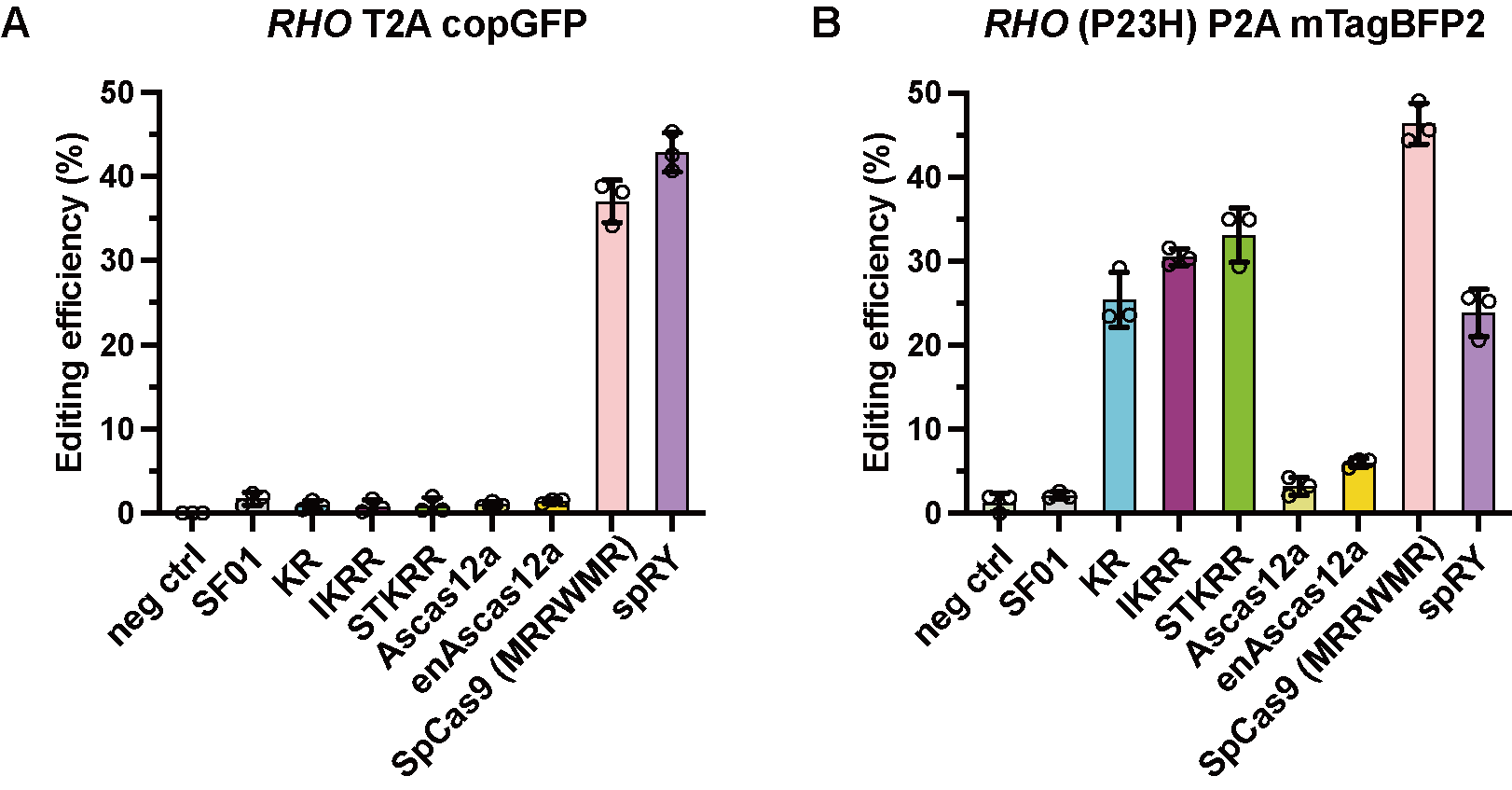


**Figure S8. Editing efficiency of SF01, KR, IKRR, STKRR, AsCas12a, enAsCas12a, SpCas9 (MRRWMR), and SpRY in piggyBac-based stable cell line co-expressing RHO-GFP and RHO P23H–BFP.**

(A) Flow cytometry analysis of editing efficiency at the *RHO* locus (AGGG PAM) monitored by GFP signal reduction. Data are shown as mean ± s.d., n = 3 independent biological replicates.

(B) Flow cytometry analysis of editing efficiency at the *RHO* P23H mutant locus (AGTG PAM) monitored by BFP signal reduction. Data are shown as mean ± s.d., n = 3 independent biological replicates.


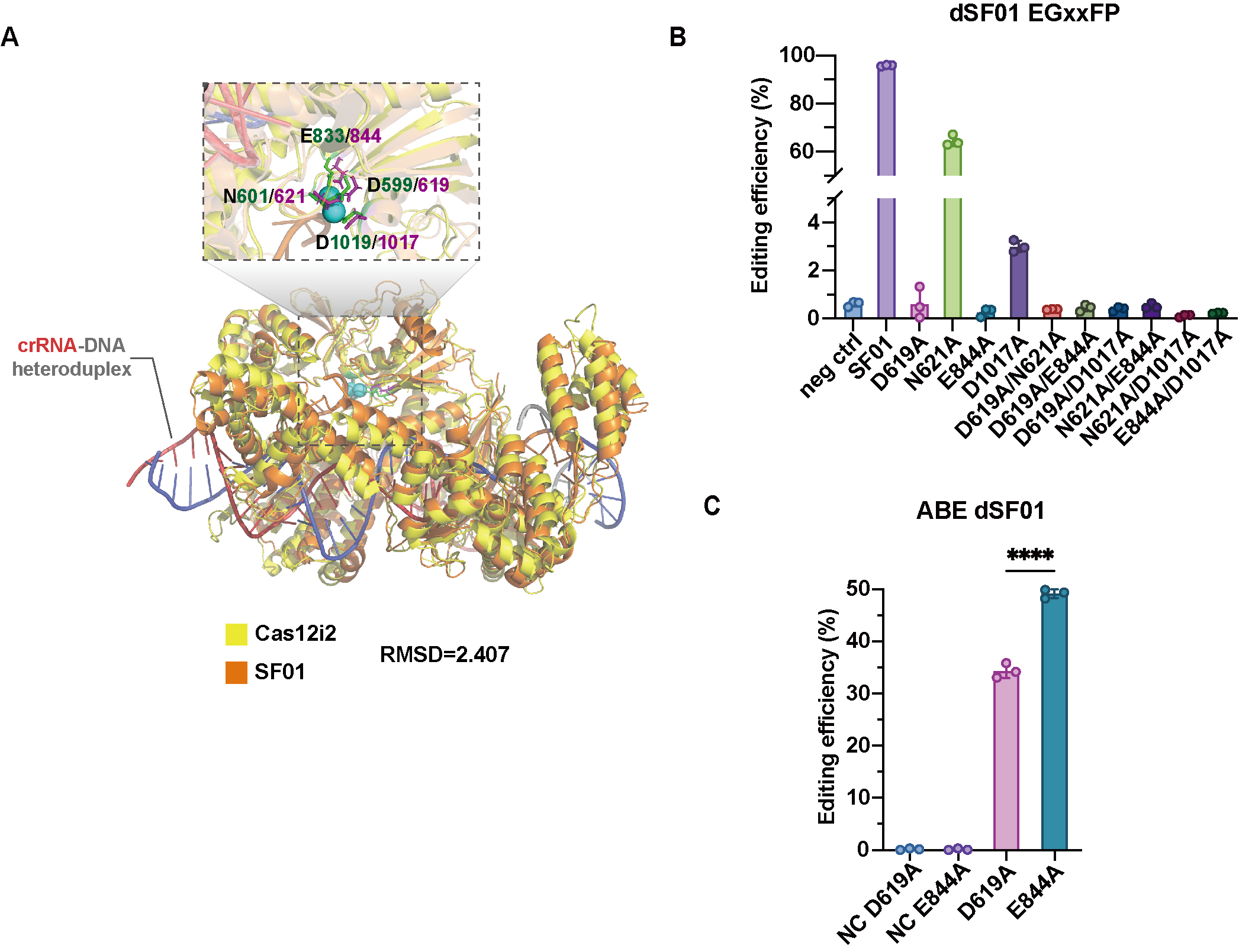


**Figure S9. Construction and evaluation of adenine base editing (ABE) activities of dead SF01 (dSF01) in HEK293T cells.**

(A) Structural alignment of SF01 (orange) and Cas12i2 (yellow), with active-site residues highlighted (green for Cas12i2, magenta for SF01) and Mg²⁺ ions shown in cyan.

(B) Evaluation of editing efficiencies of dSF01 single and double mutants using the EGxxFP reporter assay. Data are shown as mean ± s.d., n = 3 independent biological replicates.

(C) Assessment of adenine base editing (ABE) activities of dSF01 D619A and E844A variants in the ABE reporter system. Data are shown as mean ± s.d., n = 3 independent biological replicates.

Statistical significance was evaluated using unpaired Student's t-test. *****p* < 0.0001, ****p* < 0.001, ***p* < 0.01, **p* < 0.05, and ns, not significant.

Table S1. Candidate mutagenesis sites near the PAM duplex of SF01.

| No. | position | residue | domain | mutations |
| --- | --- | --- | --- | --- |
| 1 | 5 | E | WED I | 8 |
| 2 | 165 | D | Helical I | 8 |
| 3 | 166 | D | Helical I | 8 |
| 4 | 167 | V | Helical I | 8 |
| 5 | 168 | N | Helical I | 7 |
| 6 | 170 | W | Helical I | 8 |
| 7 | 171 | G | Helical I | 8 |
| 8 | 174 | S | Helical I | 8 |
| 9 | 178 | G | Helical I | 8 |
| 10 | 179 | E | Helical I | 8 |
| 11 | 180 | G | PI | 8 |
| 12 | 183 | E | PI | 8 |
| 13 | 227 | V | PI | 8 |
| 14 | 234 | S | PI | 8 |
| 15 | 235 | T | PI | 8 |
| 16 | 236 | G | PI | 8 |
| 17 | 238 | T | PI | 7 |
| 18 | 239 | A | PI | 8 |
| 19 | 240 | S | PI | 8 |
| 20 | 243 | S | PI | 8 |
| 21 | 256 | Q | PI | 7 |
| 22 | 260 | N | PI | 7 |
| 23 | 293 | D | Helical I | 8 |
| 24 | 294 | Q | Helical I | 7 |
| 25 | 295 | N | Helical I | 7 |
| 26 | 299 | Q | Helical I | 7 |
| 27 | 306 | S | Helical I | 7 |
| 28 | 453 | T | WED II | 7 |
| 29 | 456 | N | WED II | 8 |
| 30 | 457 | S | WED II | 8 |
| 31 | 458 | A | WED II | 8 |
| 32 | 460 | V | WED II | 8 |
| 33 | 462 | E | WED II | 8 |
| 34 | 494 | E | WED II | 8 |
| 35 | 495 | M | WED II | 8 |
| 36 | 582 | S | WED II | 7 |
| 37 | 597 | V | WED II | 8 |
| 38 | 599 | S | WED II | 7 |
| Total |  |  |  | 293 |

Table S2. Sequences used in this study.

| **1. Target sequences used in EGxxFP/ABE reporter system in this study** | | | | |
| --- | --- | --- | --- | --- |
| **Name** | **Sequence (5'-3')** | | | |
| T8 | ccctgttatccctaagagat | | | |
| crGFPm2 | CTCACtcatcaTGGGCCAGG | | | |
| crRHO | gctgcgtaccacacccgtcg | | | |
|  |  |  |  |  |
| **2. Endogenous target sequences** | | | | |
| Gene | Sequence (5'-3') | PAM | Name | Figure |
| *PDCD1* | cgccgggccctgaccacgct | ATTG |  | Fig.2 |
| *PDCD1* | tccccagccctgctcgtggt | CTTC | CTTC site1 | Fig.2 |
| *PDCD1* | acctgcagcttctccaacac | CTTC | CTTC site2 | Fig.2 |
| *PDCD1* | tccaacacatcggagagctt | CTTC | CTTC site3 | Fig.2 |
| *PDCD1* | gtgctaaactggtaccgcat | CTTC | CTTC site4 | Fig.2 |
| *PDCD1* | cccgaggaccgcagccagcc | CTTC | CTTC site5 | Fig.2 |
| *PDCD1* | cgtgtcacacaactgcccaa | CTTC | CTTC site6 | Fig.2 |
| *PDCD1* | cacatgagcgtggtcagggc | CTTC | CTTC site7 | Fig.2 |
| *PDCD1* | atctgcgccttgggggccag | TTTG |  | Fig.2 |
| *PDCD1* | gcacgaagctctccgatgtg | TTTA |  | Fig.2 |
| *PDCD1* | tagcaccgcccagacgactg | GTTG |  | Fig.2 |
| *PDCD1* | cagatcccacaggcgccctg | CATG |  | Fig.2 |
| *ITGB1* | aatttacaaccaattttctg | GATG |  | Fig.2 |
| *CD151* | ggtgagttcaacgagaagaa | GATG |  | Fig.2 |
| *PCSK9* | ggcaccgtcagctccaggcg | CATG |  | Fig.2 |
| *TTR* | tggccgtgcatgtgttcaga | AATG | site1 | Fig.2 |
| *TTR* | gcttctcatcgtctgctcct | GATG | site2 | Fig.2 |
| *TTR* | gtcaaagttctagatgctgt | GATG | site3 | Fig.2 |
| *VEGFA* | gacgggtgaggcggcggtgt | CATG | site1 | Fig.2 |
| *VEGFA* | aactttctgctgtcttgggt | CATG | site2 | Fig.2 |
| *VEGFA* | ccaaggtaagcggtcgtgcc | CATG | site3 | Fig.2 |
| *ITGB1* | gattggactgatcagttcag | TCTG |  | Fig.3 |
| *ITGB1* | tgtttgctcaaacaggtaaa | TGTG |  | Fig.3 |
| *ITGB1* | caatccagaaaattggttgt | AGTC |  | Fig.3 |
| *PCSK9* | ctggtggccgctgccactgc | GGTC |  | Fig.3 |
| *TTR* | ctctgccttgctggactggt | CCTC |  | Fig.3 |
| *TTR* | gggccagcctcagacacaaa | CGTA |  | Fig.3 |
| *VEGFA* | ggaagccgggctcatggacg | GCTC |  | Fig.3 |
| *VEGFA* | gcgactcggcgctcggaagc | AGTG |  | Fig.3 |
| *PCSK9* (HepG2) | tcgccttggaaagacggagg | AATG |  | Fig.4 |
| *PCSK9* (N2a, Hepa1-6) | ggcacccactgctctgcgtg | GATG |  | Fig.4 |
| *TTR* | ttagaagtccaggcagagac | TATG |  | Fig.5 |
| *TRAC* | tcaggcagtgacaagcagca | AATG | TRAC site1 | Fig.5 |
| *TRAC* | tgtcacaaagtaaggattct | AATG | TRAC site2 | Fig.5 |
| *PCSK9* (N2a) | gctgccatgggaagatggaa | AATG | PCSK9 (N2a) site1 for ABE | Fig.5 |
| *PCSK9* (N2a) | gtggggtggtgactcaccgg | GATG | PCSK9 (N2a) site2 for ABE | Fig.5 |
|  | | | | |
| **3. The sequences of crRNA synthesized chemically** | | | | |
| **Name** | **Sequence (5'-3')^1^** | | | |
| SF01 DNMT | mC*mU*mC*UAGAGAAUGUGUGCAUAGUCACACcugaugguccaugucuguuaUU*mU*mU*mUU | | | |
| enas DNMT | mU*mA*mA*UUUCUACUCUUGUAGAUCUGAUGGUCCAUGUCUGUUACUCUU*mU*mU*mUU | | | |
| T8 SF01 | mC*mU*mC*UGACCACCUGAGAGAAUGUGUGCAUAGUCACACCCCUGUUAUCCCUAAGAGAU*mU*mU*mU | | | |
| mPCSK9_SF-ATG_nu_crRNA | mC*mU*mC*UAGAGAAUGUGUGCAUAGUCACACGGCACCCACUGCUCUGCGUGUU*mU*mU*mUU | | | |
| SF01 PDCD1 | mC*mU*mC*UGACCACCUGAGAGAAUGUGUGCAUAGUCACACUagcaccgcccagacgacUgmU*mU*mU*U | | | |
| hPCSK9_aaTG SF01 | mC*mU*mC*UAGAGAAUGUGUGCAUAGUCACACucgccuuggaaagacggaggUU*mU*mU*mUU | | | |
|  |  |  |  |  |
| **4. The sequences used in cleavage assays** | | | | |
| **Name** | **Sequence (5'-3')** | | | |
| FAM ATTA F | FAM_GACGACAAAACATTAccctgttatccctaagagatTGTCTGTGGAATGCTA | | | |
| FAM CATG F | FAM_GACGACAAAACCATGccctgttatccctaagagatTGTCTGTGGAATGCTA | | | |
| FAM CCTC F | FAM_GACGACAAAACCCTCccctgttatccctaagagatTGTCTGTGGAATGCTA | | | |
| FAM AGTG F | FAM_GACGACAAAACAGTGccctgttatccctaagagatTGTCTGTGGAATGCTA | | | |
| ATTA R | TAGCATTCCACAGACAatctcttagggataacagggTAATGTTTTGTCGTC | | | |
| CATG R | TAGCATTCCACAGACAatctcttagggataacagggCATGGTTTTGTCGTC | | | |
| CCTC R | TAGCATTCCACAGACAatctcttagggataacagggGAGGGTTTTGTCGTC | | | |
| AGTG R | TAGCATTCCACAGACAatctcttagggataacagggCACTGTTTTGTCGTC | | | |

^1^: * denotes a phosphorothioate modification. m denotes a 2′-O-methyl (2′-O-Me) modification.
